# Plastic recycling plant as a point source of microplastics to sediment and macroinvertebrates in a remote stream

**DOI:** 10.1186/s43591-022-00045-z

**Published:** 2022-12-08

**Authors:** Emilie M. F. Kallenbach, Tor Erik Eriksen, Rachel R. Hurley, Dean Jacobsen, Cecilie Singdahl-Larsen, Nikolai Friberg

**Affiliations:** 1 NIVA Denmark Water Research, Njalsgade 76, 2300 Copenhagen S, Denmark; 2 University of Copenhagen, Universitetsparken 4, Copenhagen Ø, Denmark; 3 NIVA, Økernveien 94, 0579 Oslo, Norway; 4 Water@Leeds, School of Geography, University of Leeds, Leeds, LS2 9JT UK

**Keywords:** Freshwater, Size, Plastic production, Biota, Plastic waste

## Abstract

Microplastic is now ubiquitous in freshwater, sediment and biota, globally. This is as a consequence of inputs from, for example, waste mismanagement, effluents from wastewater treatment plants and surface runoff from agricultural areas. In this study, we investigated point source pollution of plastic to an upland stream, originating from a recycling plant that recycles polyethylene film in a remote area of Norway. Sediment (~2 kg) and macroinvertebrates (549 individuals in total) were sampled at one site upstream and two sites downstream of the recycling plant to study microplastic deposition and food web uptake. In total, 340 microplastic films were identified through a combination of visual and µFTIR analysis in the sediment samples. This corresponded to a concentration of 0.23 (± 0.057) items per g sediment upstream of the plastic recycling plant and 0.45 (± 0.017) and 0.58 (± 0.34) items per g downstream. The dominant plastic polymer was polyethylene, which increased significantly downstream of the plastic recycling plant. This indicates the role of the plastic recycling plant as a point source for microplastic in this catchment. Among the three sites investigated, a fairly constant concentration of polypropylene was found, indicating a diffuse source of polypropylene films across the catchment possibly relating to low-intensity agricultural land-use. Low levels of polyethylene were also observed upstream, which may be linked to either local or longer-distance atmospheric transport. Despite the considerable presence of microplastic in sediments, concentrations in macroinvertebrates were extremely low with only a single microplastic particle identified in the total of 549 macroinvertebrates—belonging to three different feeding groups—investigated. Our study suggests that: 1) microplastic pollution can be transferred to remote areas as unintended losses from recycling facilities, 2) remote areas with limited land-use pressure still have detectable levels of microplastic and 3) microplastic is only taken up by stream macroinvertebrates to a limited degree despite relatively high sediment concentrations, and thus there are no strong indications for ecological risks posed by microplastic to this ecological group at this location.

**Supplementary Information:**

The online version contains supplementary material available at 10.1186/s43591-022-00045-z.

## Introduction

Plastic is a widely used material which constitutes an important part of many consumer products. The high demand for, and use of, plastic have led to widespread microplastic pollution that is now observed across the globe [1]. Freshwater systems are the primary recipients for many sources of microplastics to the environment, including wastewater treatment plants, runoff from urban and agricultural areas and industry [2]. Both point and diffuse sources contribute to the total concentration of microplastic observed in freshwaters. Hotspots have been recorded in urban and highly populated areas [2, 3]; however, an increasing awareness of the sometimes unexpectedly high concentrations in non-urban areas is also emerging [4]. Freshwaters do not only act as a vector for the transport of microplastics to the marine environment [5] but also as a complex environmental setting, where microplastics may also accumulate and be stored [6–8]. Much focus has been placed on recycling and reusing plastics to reduce the environmental footprint associated with their production, as well as the potential risks that mismanaged plastic waste pose [9, 10]. Plastic recycling plants have therefore been established in many locations globally. However, these recycling plants are potential sources of plastic contamination to the nearby environment, including freshwater systems, if they are not strictly regulated in terms of emission control [11, 12].

Stream macroinvertebrates include benthic dwellers that live in close association with sediments, either on sediment surfaces or within the sediment, and have been widely used to assess the status of freshwater systems (European water framework directive [13, 14]). The macroinvertebrate community consists of very diverse groups which show different tolerances/sensitivities towards different stressors and some of these groups also have long life cycles, which enables accumulation of contaminants over time [15]. In addition, different species have different feeding strategies and thus may have different associated risks of exposure [16–18]. Bivalves and net spinning caddisfly larvae, among others, filter particles in suspension, whereas other groups ingest benthic organic matter, sediments and biofilm, or are predatory. Macroinvertebrates also represent an essential link to higher trophic levels in the freshwater food web and may, thus, constitute an important pathway for microplastic into the food web [19, 20]. Earlier in-situ studies have observed the occurrence of microplastic in several such species and exposure studies have likewise documented ingestion of microplastics by various benthic macroinvertebrates (for a full list of studies on microplastic ingestion by benthic freshwater macroinvertebrates see Supplementary material Table SI1). Since microplastics are within the size range of typical macroinvertebrate food items, it could be hypothesised that this organismal group could also work as a sentinel for microplastic pollution.

Exposure studies on benthic freshwater macroinvertebrates investigating their capacity to ingest microplastic and documenting their response have been carried out under laboratory conditions, including different microplastic types and exposure concentrations (see Supplementary material Table SI1). The remaining studies have focused on documenting the presence of microplastics in macroinvertebrates in their natural habitats (in-situ*)* (see Supplementary material Table SI1)*.* For exposure studies, however, it is difficult to mimic the many factors that affect uptake in nature e.g. food availability, flow velocity and species interactions. At the same time, in-situ studies might suffer from low environmental concentrations translating into limited exposure. The need for further research on in -itu uptake by a variety of macroinvertebrates has been raised [21, 22].

A range of different benthic freshwater species have been studied in-situ*,* of which the majority have been found to take up microplastic under natural conditions (22 studies, see references in Supplementary Material Table SI1). Having documented that microplastic is taken up by macroinvertebrates, a natural next step is to investigate how they respond to different environmental concentrations in-situ and how different feeding traits affect the uptake of microplastics. Anecdotal observations have indicated high levels of plastic residues within the stream sediments downstream of a plastic recycling plant in a remote upland river in the Folla valley, Norway, which provided the impetus for this study. We therefore sampled sediments and macroinvertebrates upstream and downstream of the point source of plastic pollution i.e. the plastic recycling plant. Three macroinvertebrate species belonging to different functional feeding groups were studied for plastic uptake.

*Arctopsyche ladogensis* forage using a filter-feeding strategy. The larvae construct silken nets attached to the river substrate to capture other drifting invertebrates. As the larvae grow, the net mesh size also increases. Studies from the same river network (River Glomma) showed that, in their 4th and 5^th^ instar, mesh sizes of 300 × 209 µm and 468 × 312 µm, respectively, were produced [23], hence allowing for the capture of larger prey animals as the animals grow. Studies of gut analysis also showed that late instars mainly feed on aquatic insects, such as Chironomidae, Ephemeroptera, Plecoptera, Trichoptera (even early instars of their own species), together with its own net [23]. We therefore predict that this species will capture microplastic directly in its net, and possibly ingest it. Microplastic ingestion may also occur indirectly via plastic accumulated in prey items.

The predaceous plecopteran *Diura nanseni* (Perlodidae) has a contrasting feeding strategy to *A*. *ladogensis*. *D*. *nanseni* hunts actively for prey insects on the riverbed; although, it may share many of the same prey items as *A*. *ladogensis*. Previous studies of gut analysis of *D*. *nanseni* from a West-Norwegian river has shown that early instars forage on detritus; however, late instars were entirely predaceous [24]. *D*. *nanseni* has a one-year life cycle in Norwegian rivers with emergence in spring.

*B*. *rhodani* applies a feeding strategy that combines grazing/scraping with gathering/collecting. The larvae feed predominantly on fine detritus and algae [24, 25]. *B*. *rhodani* has a flexible life cycle strategy and produces a variable number of generations per year depending on environmental conditions, such as water temperature and food availability [25].

We expect to find high concentrations of microplastic in the sediments downstream the recycling plant – compared with average Scandinavian rivers—and thus to find sufficiently high exposure conditions for macroinvertebrates to interact with and ingest plastics. This will provide us with unique information about ingestion by these organisms and how different feeding guilds affect proneness towards ingesting plastic in-situ at high, but environmentally relevant, concentrations. Furthermore, it will give insight into the interplay and relation between microplastic in sediment and microplastic in macroinvertebrates.

In this study, we only analysed microplastic films and fragments, as these represent the material associated with the plastic recycling plant. Thus, we excluded spherical particles and fibres. Particles with a low thickness (up to approx. 30 µm) compared to the other two dimensions were categorised as films, whereas fragments were defined as irregular shaped particles with a larger thickness than films.

We hypothesise that 1) the plastic recycling plant constitutes a point source of plastic pollution to the River Folla, 2) microplastic concentration varies between sites upstream and downstream of the point source in both macroinvertebrates and sediment, 3) microplastic concentrations in sediment and organisms decrease with distance downstream from the point source and 4) microplastic concentrations in individuals of macroinvertebrates vary in accordance with their functional feeding group.

## Methods

### Sampling strategy, study area and sampling time

**Fig. 1:**
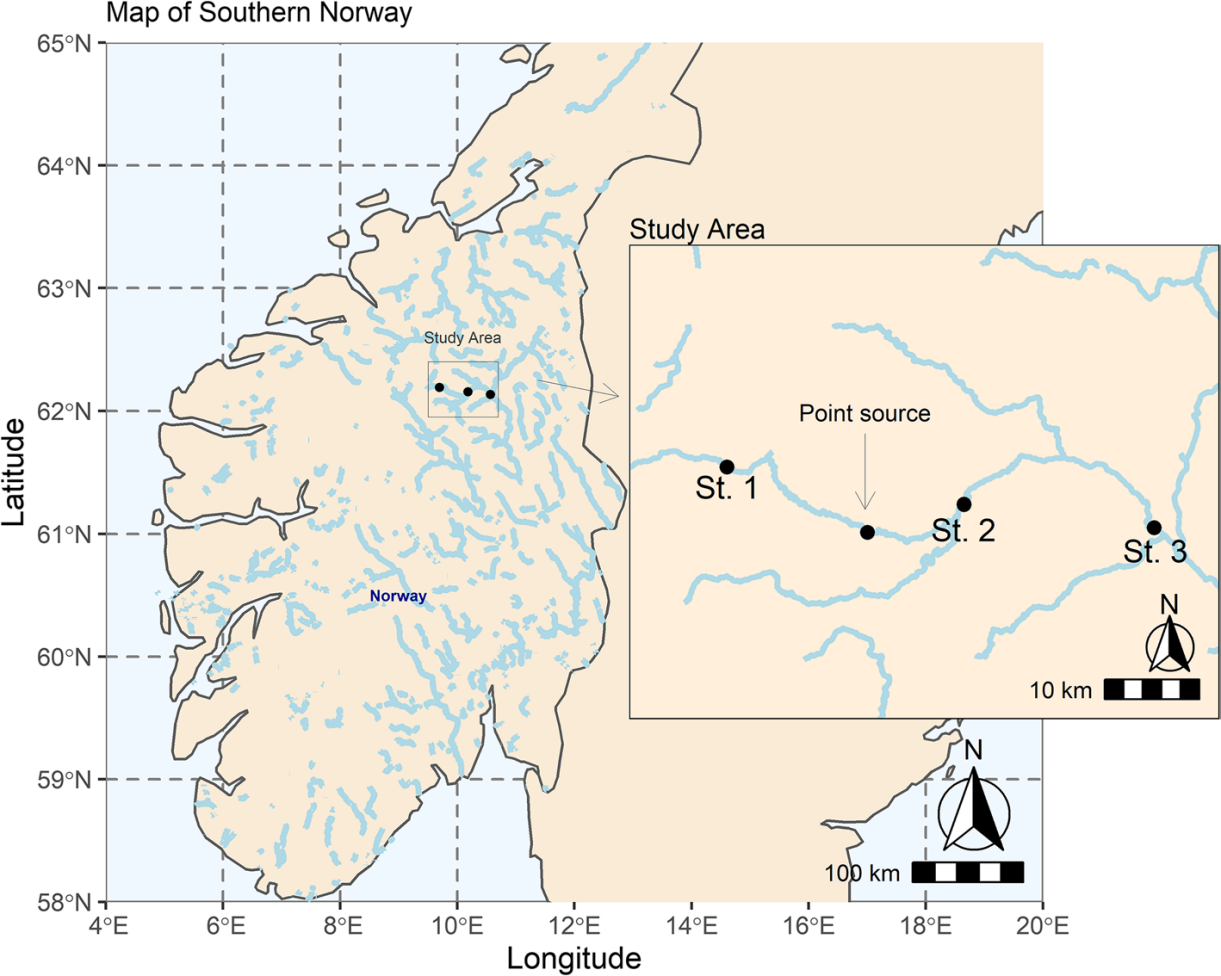
Map of Southern Norway. The zoomed map shows sampling sites and the location of the point source of plastic contamination

The River Folla is in a mountainous area in Innlandet county, central Norway (Fig. 1). The catchment covers ∼2,432 km^2^, with a minimum and maximum elevation of 477 and 1,851 m, respectively. The average mean water discharge in the River Folla is ∼22 m^3^/s (Dølplass station, 530 m above sea level, period 2011 – 2021). Only approximately 1.8% of the catchment area is cultivated (agriculture and farming) and urban areas make up only a marginal part (0.04%). The River Folla runs through the village Folldal. Folldal municipality has two small wastewater treatment plants discharging into the river. One is placed in the Center of Folldal (capacity: 2,000 persons) and one is 15 km away (capacity: 550 persons). Both include filtration of the outflow, which, after sludge has been separated away, is gathered in sedimentation basins. In Folldal, there is a plastic recycling plant that recycles polyethylene film, situated on the bank of the river. The plant recycles polyethylene film primarily from agricultural use e.g. from the wrapping of hay bales. The plastic films are rinsed with water, and the resulting wastewater is first fed into sedimentation tanks, and thereafter discharged into the River Folla. The recycling plant has permission to receive 26,000 tonnes of plastic waste and produces 16,000 tonnes of recycled polyethylene pellets per year (in 2017 the production was 15,000 tonnes and they received 20,000, and therefore the permissions was increased). There is no information about how they dispose of the material – corresponding to 10,000 tonnes—that is not recycled. They receive approximately 50% of all plastic film generated in Norway but do also receive plastic produced in Europe. Since storage of plastic waste is observed on outside facilities, up to 2,000 tonnes per year, aerial dispersion may also be a route of transfer to the environment.

Sampling was carried out between the 27^th^-28^th^ of April and on the 26^th^ of June 2021. An initial field campaign was carried out in April and September 2019 to select appropriate sites and assess available species for analysis. Species of macroinvertebrates collected from the first sampling period in April 2019 were retained for gut analysis.

### Sampling sites

Samples of benthic macroinvertebrates and sediments were collected from three sites in the River Folla (Fig. 1): Deplflyin (St. 1), Grimsbu (St. 2) and Gjelten bru (St. 3) situated at ∼800—480 m above sea level. St. 1 is located upstream of the recycling facility, while St. 2 and St. 3 are both downstream. Characteristics of the studied rivers sites and catchments is provided in Tables 1 and 2. Data on the catchment size, distance from river mouth and distance from the plastic recycling plant are taken from the Norwegian national mapping service [26]. The spatial extent of each site monitored in the River Folla was 25 – 30 m. All sites were relatively similar in terms of substrate and velocity (Table 1).

**Table 1:** Catchment and stream characteristics for each of the three sampling sites. Substrate size was logarithmically transformed to Krumbein scale, *phi* units (φ)

Site	Latitude Longitude	Catchment size (km^2)^)	Distance from plastic recycling plant	Length from river origin (km)	River width (m)	Yearly discharge (Mm^3^/year)	Slope	Boulder (%)	Cobble (%)	Coarse gravel (%)	Coarse pebbles (%)	Small and medium pebbles (%)	Sand (%)	Phi (φ)
St. 1	62.1892, 9.6988	270	22.7 km upstream	42.7	9	117.8	8.3	1	10	55	14	15	5	-5.9
St. 2	62.1537, 10.1807	1197	13.9 km downstream	79.3	48	522.3	8.9	5	25	42	15	10	3	-6.6
St. 3	62.1317, 10.56768	2128	43.4 km downstream	108.8	50	928.5	9	1	10	60	10	15	4	-6.0

**Table 2:** Percentage of different land use in the catchment of each of the three sampling sites

Site	Swamp %	Lake %	Alpine %	Unclassified %	Agriculture %	Forest %	Urban %
St. 1	5.5	2.0	55.8	13.5	0.8	22.4	0
St. 2	4.5	1.1	53.1	9.1	2	29.9	0.1
St. 3	5.9	1.7	53.8	9.1	1.9	27.5	0

### Sampling of macroinvertebrates

Three field campaigns were initiated in the River Folla for the purpose of studying macroinvertebrate compositions in the river network, selecting study organisms and gut analysis. The study sites were visited in late April 2019, September 2019 and the beginning of May 2021. Among the species identified in the initial field campaign in April, three species were chosen for this study based on their availability at all sites, the size of the organisms and the corresponding feeding trait (Full species lists can be found in supplementary material Table SI2). The three different species chosen for analysis of microplastic content were: 1) the trichopteran *A. ladogensis* (Arctopsychidae; Kolenati, 1859)*,* 2) the plecopteran *D. nanseni* (Perlodidae; Kempny, 1900) and *3*) the ephemeropteran *B. rhodani* (Baetidae; Pictet, 1843–45).

Macroinvertebrates were collected for plastic analysis using kick samplers (250 µm mesh net). Material from the kick samplers was transferred to white trays (polypropylene) and the target organisms were picked and transferred to prewashed glasses. Kick sampling was repeated until a minimum of 21 individuals of each species were found at each site. In the present study, specimens of *A*. *ladogensis* with body sizes of ∼2 cm were collected from the different sites to represent 4^th^ and 5^th^ instar larvae. We targeted only late instars (4th and 5^th^) of *D. nanseni* known to be entirely predaceous [24] and for *B. rhodani*, we targeted predominantly large-sized nymphs. The samples were frozen immediately after sampling and were kept frozen until analysis. Number of individuals sampled at each site, can be found in Supplementary material Table SI3.

### Sampling of sediment

Sediment was collected from eight different locations within each site: four samples were collected at coarse substrate locations and four samples were collected at fine substrate locations. This was undertaken to capture the potential intra-site variability in sediment storage and microplastic accumulation. Each site was defined as a reach extending approximately 30 m in length, and across the full river width. However, the water depth and velocity did not allow sampling from the entire reach, but sites were selected to get as representative a sample of the reach as possible, and the three sampling sites were very similar. Approximately 5 cm of the top sediment was collected using shovel sampling within a metal frame of a fixed quadrat sampler (Surber sampler; 14 × 14 cm; KCC, Denmark). The Surber sampler was placed on the riverbed in an inverted position, with the sampling net facing upstream. The Surber sampling net (250 µm) was pulled upwards, closing the frame of the device facing upstream, to prevent disturbances from river water and sediments during the collection of sediments. In addition, for the same purpose, the person conducting the sampling was positioned in front of the sampler. The eight sediment samples from each site, corresponding to the following masses St. 1: 744.25 g, St. 2: 731.2 g, St. 3: 546,2 g, were decanted and pooled for each site in glass containers with a metal lid and stored cool (5 °C) until analysis.

### Grain size composition of the substratum

Sediment grain size was visually assessed from each sampling site based on Wentworth (1922) and as described in [27]: sand (0.064—2 mm), small and medium pebbles (2.1—16 mm), coarse pebbles (16.1—64), cobbles (64.1—256 mm), and boulder (> 256 mm). Substrate size was logarithmically transformed to Krumbein scale with *phi* units (φ), and a score was calculated for the average of the values based on the relative substrate composition. The following *phi* units were adopted for these calculations: silt and clay = 8.89; sand = 2.97, small and medium pebbles = -3.24; coarse pebbles = -5.24; coarse gravel = -7; cobble = -8.5; boulder = -10. Positive *phi* scores are therefore associated with finer sized substrate particles and negative numbers with coarser particles.

### Laboratory analyses

#### Quality control quality assurance

Risk of contamination is reduced compared to non-target microplastic analyses, as we were only assessing the occurrence of two colours (white/transparent) and two particle shapes (films and fragments) in this study. Normally fibres are found to dominate blank samples [28]; however, fibres are not produced by the recycling plant and were not taken into consideration in the present study. Nevertheless, precautions were still taken to avoid sources of contamination. All reagents used for cleaning and sample processing were first filtered through a VacuCap 90 filter unit (0.2 µm super membrane, Pall Corporation, Cheltenham, VIC, Australia) and all equipment was washed three times with filtered demineralised water prior to use. All lab work with macroinvertebrates was carried out in a fume hood while wearing cotton lab coats, cotton or wool clothing and nitrile gloves to minimise contamination. The fume hood and all laboratory equipment were washed twice with filtered demineralised water and once with filtered 76% ethanol before commencing work on the samples. Procedural blanks were carried out for every third macroinvertebrate sample and a total of three blanks spaced out evenly between the sediment samples were included. Only reagents, and no biota or sediment, were added to these blanks and they were processed following an identical procedure to the samples. As the plastic treatment plant recycles films, only white/transparent films and fragments were counted in the blanks.

Microplastic recovery tests for macroinvertebrate samples were made by adding 4 red polyethylene terephthalate (PET) fibres (200–700 µm) and 4 tyre fragments (250 µm) to 6 samples from the different sites and with different organisms. Microplastic recovery tests for sediment samples were made adding 10 polyethylene beads (300 µm) and 10 PET fibres (200–700 µm). Reference polyethylene film particles of the right size and shape were not available at the time of the study, and therefore a suite of different particle types was used to establish a more general recovery efficiency. The size of the spiked particles represents the availability of reference material in the laboratory at the time of sample processing.

Processing of sediment was carried out in a laminar flow bench in a laboratory specially designed and exclusively used for microplastic analysis. The laboratory consists of a positive pressure room with HEPA-filtered air input (class H13). Rigorous contamination reduction measures are in place, including regular cleaning, use of cotton lab coats and natural fibre clothing (cotton scrubs) and a decontamination process upon entry into the laboratory. Atmospheric blanks are taken routinely to ensure that contamination in the laboratory is maintained at an absolute minimum.

Limit of detection (LOD) and limit of quantification (LOQ) were calculated based on the method described in [29].

#### Macroinvertebrates

Verification of correct species identification was first controlled using a microscope (Olympus SZX10 stereomicroscope; 20 × magnification) in the laboratory. Organisms were thawed at room temperature, rinsed with filtered deionised water and checked for microplastic attached to the external body parts and dried at 40 °C for 48 h in an incubator, covered with aluminium foil. After drying, each species of macroinvertebrates was pooled into three replicates (7–43 individuals per replicate) and weighed on a Dual Range XS105 scale (accuracy = 0.00001 g, Mettler Toledo, USA). The length of ten individuals of each species, sampled the previous year, were measured.

A combination of H_2_O_2_ and chitinase was applied following the procedure described in Kallenbach et al. [30]. Briefly, macroinvertebrates were transferred to Erlenmeyer flasks and 30% H_2_O_2_ was added. The samples were incubated with magnetic stirring at 40 °C for 24 h. Thereafter, chitinase solution was added and the samples were incubated at 37°C with magnetic stirring for 24 h. Finally, the samples were vacuum filtered through Whatman GF/C-filters (47 mm, pore size 1.2 µm) using a glass Büchner filtration device.

The guts from *D. nanseni* sampled in spring 2019 were dissected following recommendations from [31]. *D. nanseni* were cut open on the ventral side and the gut was removed. The gut was spread/squashed out on a glass slide and visual estimations using a stereoscope were made on how full the guts were and the relative proportions of biota, amorph material and inorganic material. The gut and gut content were both filtered onto pre-weighed GF/C-filters and dried at 40 °C for 2 days. The dry weight of the gut content was recorded.

#### Sediment

Glass jars containing the sediment samples were covered with tin foil and freeze dried for 5 days. The sediment was thoroughly mixed and three subsamples of three replicates of 30 (± 2) g each were taken and transferred to individual Falcon tubes (n = 9, three per replicate). The remaining sediment was weighed.

Density separation was carried out by adding a saturated Sodium Iodide (NaI) solution (density = 1.79 g/cm^3^) to the Falcon tubes. The material was thoroughly mixed into suspension in the density solution and allowed to settle out completely. The supernatant was passed through a 75 µm sieve and the retained material (>75 µm) was transferred to an Erlenmeyer flask. This process was repeated a second time to enhance the recovery of microplastic from the sample. The >75 µm material from the first extraction was then subject to peroxidation using H_2_O_2_ to reduce the organic content. Briefly, 30 ml of 30% H_2_O_2_ was added to the sediment samples. An ice bath was prepared, and the Erlenmeyer flasks were placed in the ice bath if the reaction became too violent or if the temperature exceeded 40 °C. Temperatures higher than this can damage the microplastic. The samples were left in a laminar flow cabinet for 24 h, and the remaining material was vacuum filtered onto Whatman GF/A-filters. The >75 µm material from the second extraction was filtered directly onto GF/A-filters.

#### Visual and chemical characterisation

**Fig. 2:**
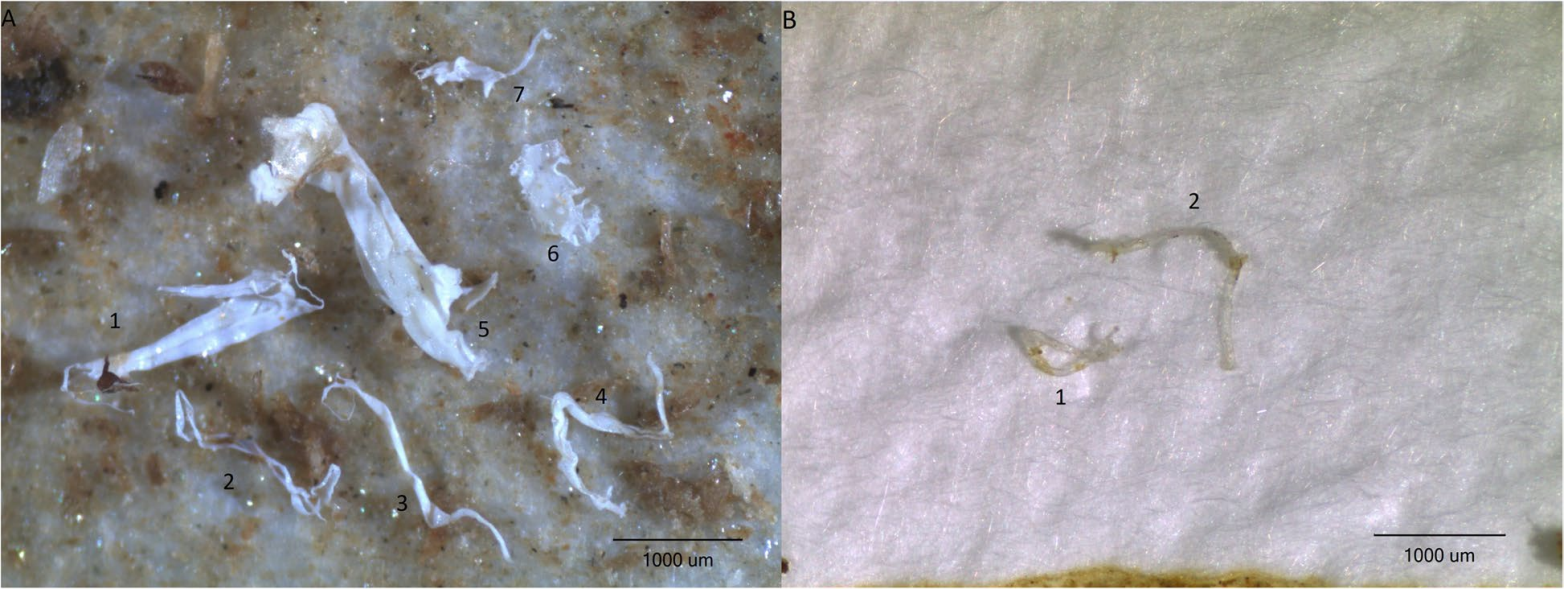
Examples of **A** films and **B** fragments

Visual detection of particles was carried out using a stereomicroscope (Nikon SMZ 745 T at 20 × magnification, lower size limit 50 µm for macroinvertebrates and 75 µm for sediment). This defined the size of microplastic targeted for analysis (sediment 75–5,000 µm and macroinvertebrates 50–5,000 µm). All suspected particles that met the predefined criteria (films and fragments, white or transparent in colour) were photographed using an infinity 1 camera. Films were defined by their low thickness (up to approx. 30 µm), while the term fragments was used to denote thicker particles (see Fig. 2 for examples). Particles were manually picked using microforceps, transferred to a diamond compression cell (Perkin Elmer DC-3) and analysed for polymer composition on a Perkin Elmer Spotlight 400 µFTIR at the NIVA Microplastic lab, Oslo, Norway. µFTIR analysis was performed in transmission mode, with a spectral resolution of 4 cm^−1^ across a spectral range 4,000-600 cm^−1^. Each spectrum was composed of a total of 2 co-scans. Every time the diamond compression cell was loaded onto the FTIR, a background measurement was taken. In total, 17 and 370 suspected particles were measured on the FTIR for the macroinvertebrates and sediments, respectively, representing 100% of the suspected microplastic particles in the macroinvertebrate samples and all (minus three that got lost) found in the sediments. One single particle, representing all of the found particles in the blanks, was also analysed.

Particles that were confirmed to be microplastic by FTIR analysis were then measured for the maximum and minimum Feret’s diameter, based on photographs taken during the visual identification step (Infinity Analyze v. 6.5.4 software, calibrated using a measurement standard).

All spectra from particles both macroinvertebrates and sediment were compared to an open microplastic reference library [32] in the Spectrum10 software (v. 10.6.2.1159). Spectra from sediment all had a match score >70% and matches were controlled manually to verify the match result.

## Results and discussion

### Quality assurance and quality control

A single polypropylene particle (film, transparent, 537 × 32 µm) was found in the one of the blanks included alongside the sediment samples. The origin of the contamination could not be identified with certainty. Based on this single particle, an LOD and LOQ was calculated, corresponding to 1.75 and 5.05, respectively.. This means that a minimum of 1.75 particles per sediment sample is needed to avoid false positives and that 5.05 particles per sediment sample is needed per sample to be able to be able to quantify limits on accuracy and for assessing the results to be reproducible. Due to the high concentrations in the sediment, these requirements were met. As only a single particle was found, the results were not corrected for this blank contamination.

No particles were found in the blanks from the macroinvertebrates, so an LOD and LOQ could not be calculated for these. Therefore, no further corrections for blanks were carried out.

The lowest detectable size was imposed by the pore size of the sieve (for sediment: 75 µm) and the lowermost size at which microplastic can reliably be picked with microforceps (for macroinvertebrates: 50 µm).

The average recovery for the macroinvertebrate test was 70.8% for the PET fibres and 79.1% for the tyre fragments. For the sediment test, the average recovery was 70% for the PET fibres were and 90% for the polyethylene beads. The variation in recoveries corresponds with generally lower recoveries for fibrous microplastic, which has also been observed in other studies[33–35]. Recovery rates were not used to correct data.

### Concentrations of microplastic in sediment

**Fig. 3:**
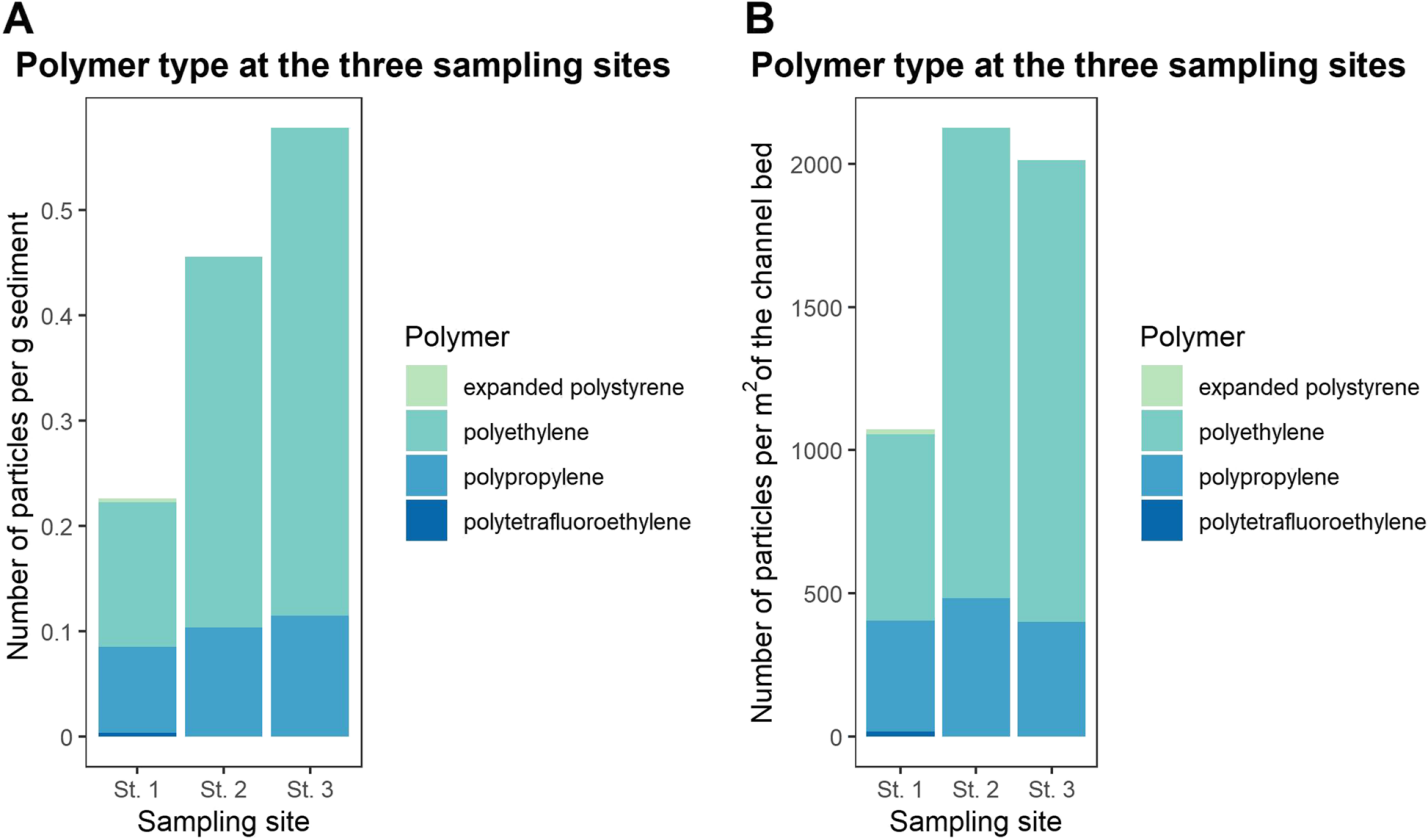
Concentrations of expanded polystyrene, polyethylene and polypropylene at the three sites reported as **A** microplastic particles per g sediment and **B** microplastic particles per m^2^ channel bed

**Fig. 4:**
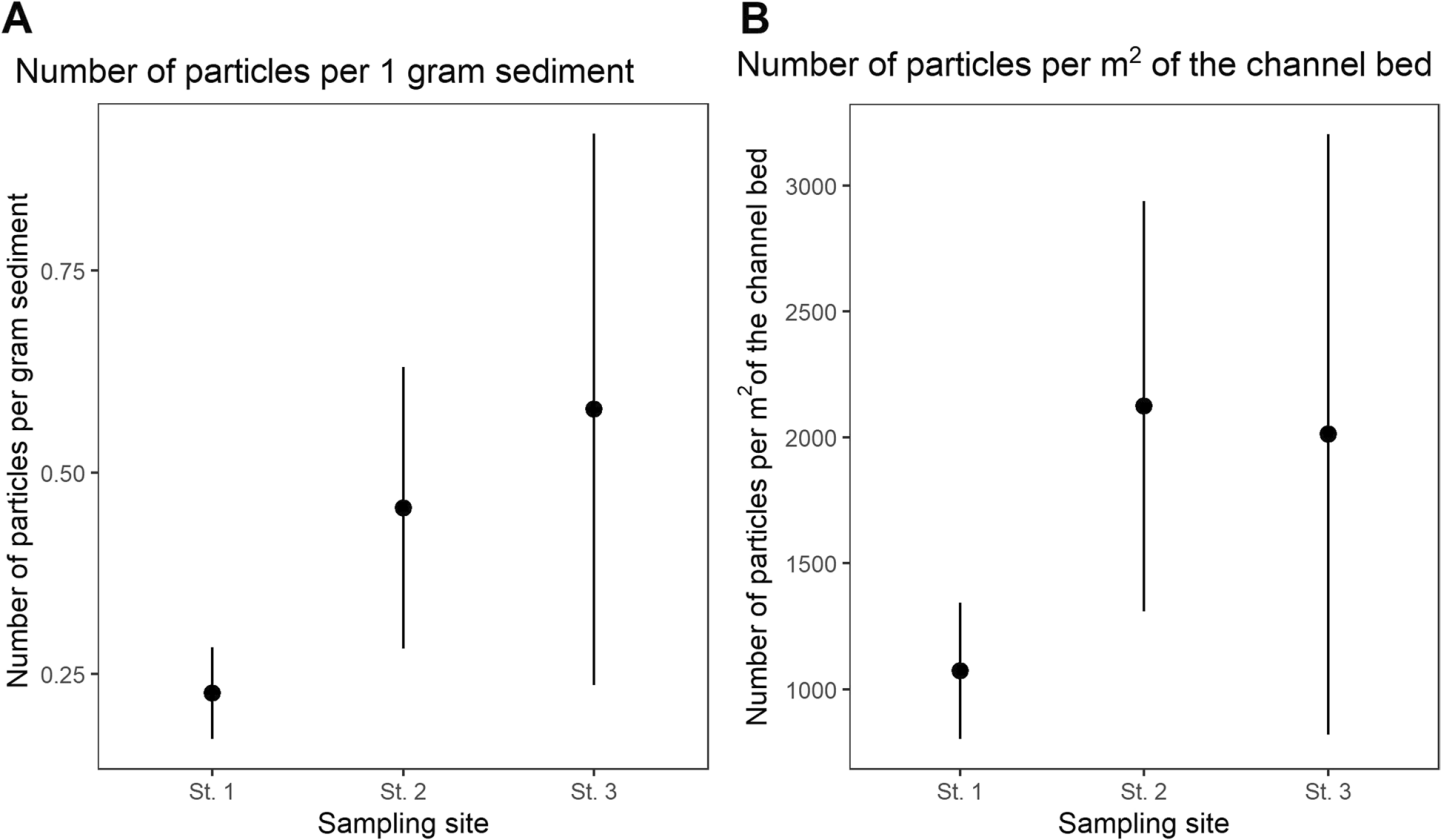
Average concentrations of microplastic when reported **A** per g. dry weight and **B** per m^2^. Error bars show S.D.s

In total, 370 particles were identified as suspected microplastic during the visual identification step, of which 367 were analysed on the µFTIR (due to small losses during sample handling). Twenty-seven particles were identified as being non-plastic, for example cellulose, wood, chitin, down, fur, kapok, jute, salt and algae, leaving 340 particles that could be identified as plastic polymers. All other particles were composed of polypropylene and polyethylene (Fig. 3). A single particle each of polytetrafluoroethylene and expanded polystyrene were found in the upstream site but not downstream of the plant. The sources of these could not be determined or linked to activities in the catchment. Notably, the levels of polypropylene pollution were relatively similar among the three sites, whereas polyethylene presented a more varied concentration following the point source (Figs. 3 and 4).

The total microplastic concentration at the upstream site was the lowest of the three sites when reported as both per area and per weight: 1,072.4 (± 1270.6) items per m^2^ or 0.23(± 0.06) items per g sediment. The concentration of microplastic in the sediment was higher just downstream of the recycling plant, with a concentration of 2,124.4 (± 814.3) items per m^2^ or 0.46 (± 0.17) items per g. Furthest downstream from the plastic recycling plant (St. 3), the highest concentrations were found when reported by mass –0.57 (± 0.34) items per g. However, when reported by area, the average concentrations were lower compared to St. 2—2,012.64 (± 1,192.3) items per m^2^ (Fig. 4) (see Table 3 for number of particles and concentrations found in each replicate at the three sites).

**Table 3:** Number of particles per replicate, grams of sediment analysed per replicate and at each site, particles per gram sediment in the three replicates and total amount of sediment sampled at the three sites

	Number of particles in Replicate 1	Number of particles in Replicate 2	Number of particles in Replicate 3	Sediment analysed per replicate per site	Particles per gram sediment in the three replicates	Sediment sampled in total per site
St. 1	16	26	19	90 g per replicate (270 g per site)	0.18 MP/g 0.29 MP/g 0.21 MP/g	744.3 g
St. 2	34	30	59	90 g per replicate (270 g per site)	0.38 MP/g 0.33 MP/g 0.66 MP/g	731.2 g
St. 3	87	40	29	90 g per replicate (270 g per site)	0.97 MP/g 0.44 MP/g 0.32 MP/g	546.2 g

The observed discrepancies between mass and area reporting units from the sampling sites indicates different processes related to sediment storage and microplastic accumulation. At St. 2, microplastic is released continuously from the plant and spread in the sediment resulting in high concentrations per area. Overall, these concentrations are less stable compared with depositional areas, e.g. at St. 3, where microplastic accumulate over time and hence has higher concentrations per mass. Less stable areas at St. 3 will not contain as much, so overall the area-based particle concentration is lower. This sheds light on the need to report microplastic data in multiple reporting units – wherever possible – to capture the full picture of microplastic pollution dynamics occurring at a given location. This can be used to assess whether higher concentrations at a site also translates into there being more microplastic particles. Different patterns provided by the different datasets can allude towards hydrogeomorphic processes that lead to differing deposition/mobilisation and sorting of microplastics in bed sediments. All the sites were relatively similar in terms of sediment grain size (Table 2); however, areas characterised by finer grained sediments could be expected to be associated with higher concentrations of microplastics, since the hydrodynamic processes for settling of sediment are similar for microplastics e.g. low flow velocity [36, 37]. It is also notable that the two dominant polymer types observed in this study are theoretically buoyant in freshwaters (densities <1.0 g/cm^3^). Many studies have already observed such low-density polymer types in freshwater sediments (e.g. [38–40]); yet, it is worth remarking that the specific processes and associated spatial and temporal scales governing the sedimentation of low-density polymers are not yet fully elucidated. Further research is required to more explicitly examine the processes that influence microplastic accumulation and mobilisation and entrainment, with reference to different particle morphologies and densities.

Several studies have reported that microplastic concentrations show a pattern of decreasing contamination downstream of a point source [2, 36, 41, 42]. This trend is observed in the current study when considering particles reported by unit area, but not by unit mass (Fig. 4). This again highlights the need to consider multiple reporting units but also indicates that typical patterns may not always occur. For example, Gallitelli, Cesarini [43] reported that microplastic in riverine sediment did not vary with distance from the source. They suggest that the variation in plastic concentration is instead explained by displacement energy. Likewise, a complex pattern of concentrations was reported for microplastic in channel bed sediments in Manchester, UK [6, 44]. Flooding was proposed as an important mechanism controlling the mobilisation and transport of microplastic; however, the studies also reveal how concentrations can vary across spatial and temporal scales. Further research is required to predict spatial distributions of microplastic in riverine sediments, in response to a range of different sources, for example through modelling approaches.

A total of 16 studies report microplastic concentrations for channel bed sediments in European riverine systems (supplementary material Table SI4). The concentrations observed in this study are comparable to or higher than those presented in studies of other relatively remote streams [45–48], and comparable to concentrations in a plastic production area in China [12]. We have a defined point source, which is reflected in the increase in concentration between St. 1 and St. 2; however, we also detected higher concentrations than expected upstream of the source, especially taking into consideration that other studies typically report the full range of plastic polymers, shapes and colors. These results highlight that even though remote regions are considered to have little spatial variation in microplastic concentrations, spatial variation and point sources for plastic contamination do exist [49].

**Fig. 5:**
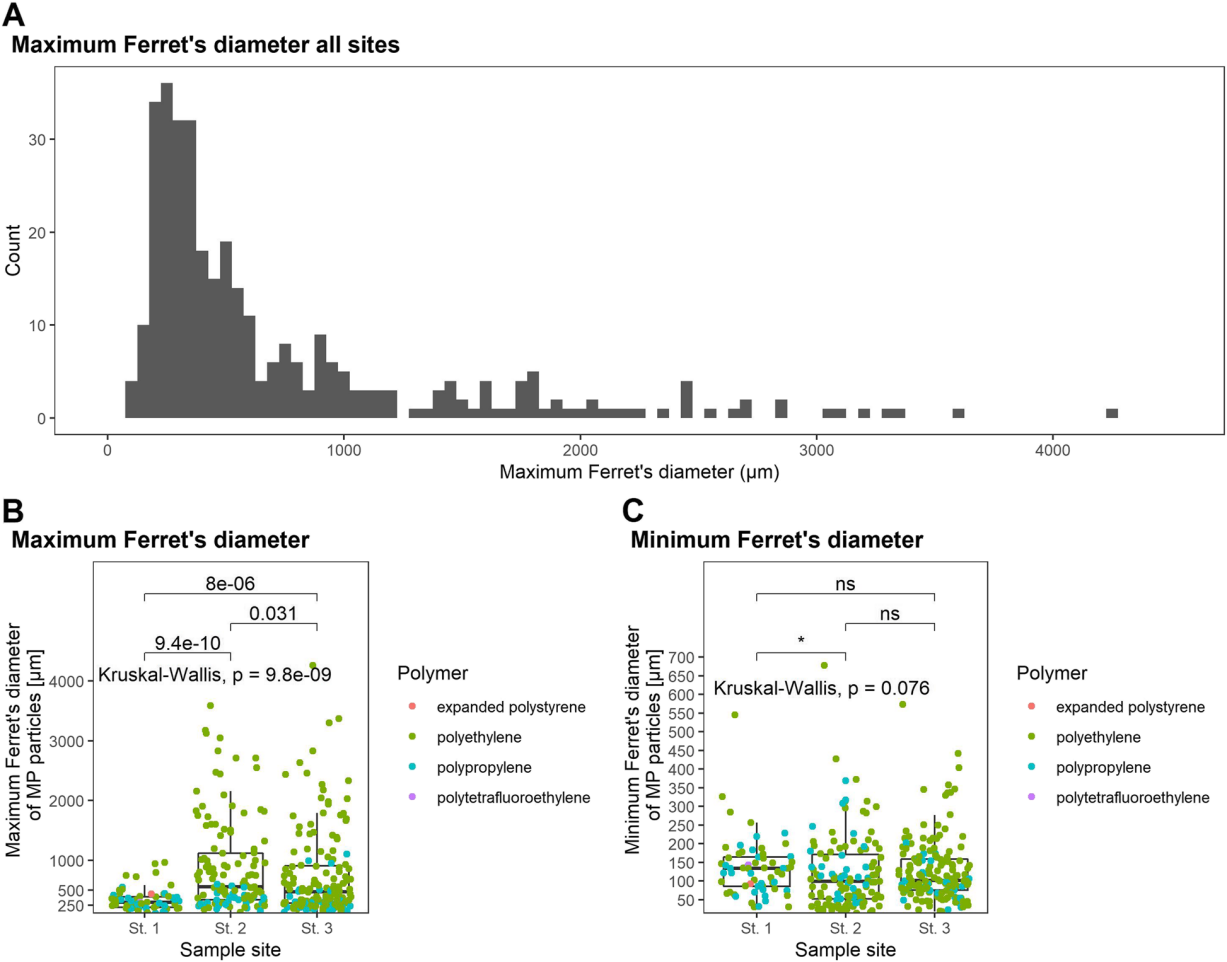
Size of microplastic in sediment. **A** Maximum Ferret’s diameter (µm) of particles of all sites, **B** maximum Ferret’s diameter (µm) of microplastics at each of the three sites and **C** Minimum Ferret’s diameter (µm) of microplastics at each site. Asterix indicate level of significance: ‘ns‘ = not significant, ‘*‘ = 0.05

The increase in the concentration of polyethylene observed between St. 1 and St. 2 clearly indicates that pollution is introduced by the point source i.e. the plastic recycling plant (Fig. 3). What is less clear is whether the concentration of polyethylene upstream, at St. 1, derives from wind born plastic from the plastic recycling plant or if it is from alternative upstream sources. It is notable that the microplastic found at St. 1 are smaller in size (Fig. 5). This could indicate particles that are preferentially transported by wind, or that they are from a different source. The theory of an additional source is supported by the polypropylene data – the particles at St. 1 are of a similar size and concentration to the PE. Yet, it is not possible to conclude on this from the current data.

Both polypropylene and polyethylene are used for agricultural purposes in Norway, for example plastic mulching and storage of crops or hay. Even though agriculture makes up a small percentage of the catchment land use (Table 1), plastic films from agriculture have been observed next to the river at St. 1 (Supplementary material Figure SI1). Plastic waste from agriculture may be transported to the river via air or surface runoff. Yet, the concentrations of polypropylene remain stable through the sites both when reported by unit mass and when reported per stream bed area, which could point towards a continuous diffuse source of this polymer. Further investigation is required to unpick additional point and diffuse sources of microplastic pollution in the catchment.

No particles below 110 µm were found in the sediment samples, despite a lower limit of detection of 75 µm. It is possible that the color of the particles – white or transparent – could have complicated the visual identification; however, this work was carried out by fully trained and experienced laboratory technicians. One possible explanation for the lack of small particles in these sediments could be that only relatively large particles are released by the recycling plant. This is in agreement with the size of microplastic found in sediment from a plastic production area in China [12]. The downstream sites were still reasonably close to the point source (St. 3 was situated 43.4 km downstream from the plant); in-situ fragmentation processes capable of generating smaller particles may not be significant at this spatial scale, especially if particles are conveyed efficiently downstream. Alternatively, small particles may not be retained in the sediments at these sites. This is supported by theoretical modelling exercises performed for freshwater sediments, which reveal that small microplastic particles are preferentially entrained [50]. This, however, is likely to vary between stream systems and sites, depending on hydrological variables, polymer density, the occurrence of storm events and sampling time. In particular, the low-density polymers that dominate at these sites may further support the increased likelihood for small particles to remain in suspension for longer distances from the input from the recycling plant, or other catchment sources.

Microplastic particle size varied between the sites (Fig. 5B). Particles at St. 2 and St. 3 were significantly larger than at St. 1, and particles at St. 2 were slightly significantly larger than at St. 3 (Fig. 5B). The Maximum Ferret’s diameter of the particles illustrates an exponentially decreasing curve from 300 µm to 3,600 µm^2^. However, below 200 µm, the frequency drops (Fig. 5A). A similar pattern has been reported in other studies of microplastics in freshwater sediments [2, 6, 51]. Thus, the particles available for digestion by the macroinvertebrates are relatively large.

### Microplastic in macroinvertebrates

In this study, a total of 549 macroinvertebrates were analysed for microplastic content, and a further 30 individuals of the species *D. nanseni* were specifically analysed for gut content. In total, 16 suspected microplastic particles from the 549 digested individuals were further characterised using µFTIR; yet, only one polypropylene and one polyester particle were confirmed to be microplastic (the polyester particle was excluded from the dataset as it did not meet the target criteria for this study). No polyethylene particles were found in any of the macroinvertebrate samples.

This indicates that macroinvertebrates, which constitute an important link to the rest of the stream food web, and are abundant at these sites, did not appear to interact with the microplastic present in the sediments.

Other studies investigating microplastics in macroinvertebrates report that the organisms contained microplastic (e.g. [52–54]). This contrasts with the present study. For *B. rhodani*, this might be explained by their relatively small body sizes; however, the plastic found in the river sediments were within the size of their preferred food items <1,000 µm [55]. *D. nanseni* and *A. ladogensis* are both predators with two different predatory strategies. These might be capable of distinguishing between plastic and prey of nutritious value; however, they are still prone to indirect ingestion of microplastic from their prey. From the dissections of the gut of *D. nanseni*, it was clear that their guts were full of animal material exclusively, illustrating that highly nutritious food were available at these sites. This is central, as studies have shown that microplastic ingestion can depend heavily on food availability, with less microplastic ingested with a higher availability of natural food sources [52, 56]. Extending beyond macroinvertebrates, results comparable to this study have been reported by [12], who observed no particles in two out of three investigated fish species in a plastic production area, although the authors were not able to conclude why this was so.

Several factors may affect the uptake of microplastics by macroinvertebrates. For example, we chose to sample during the spring, when the macroinvertebrates are actively feeding to sustain their metabolic needs with the increasing temperature. Studies using multiple seasons may be able to reveal other patterns in plastic uptake due to temporal variations in river metabolic processes and variation in macroinvertebrate feeding patterns [51]. Studies from the River Glomma, in the same river network, showed that late instars of *A*. *ladogensis* increased their gut content of animal prey in late spring, possibly to ensure a high calorie food source to prepare for pupation and emergence [23]. This indicates that the studied macroinvertebrate species have the potential to discriminate microplastic from food of higher nutritional value.

Microplastic size is also expected to be an important factor affecting uptake. In exposure studies, a preference towards smaller particles has been reported: Silva et al*.* 2021 [57] found that the dipteran larvae *Chironomus riparius* primarily ingested microplastic <75 µm when exposed to particles in the size range 32–500 µm. This is in alignment with the size of food that they would ingest under natural conditions. Another study conducted by Redondo-Hasselerharm et al. 2018 [16] found that the average size of microplastic ingested by *Gammarus pulex* was 58 µm when exposed to particles ranging between 16–165, and that particles >165 µm made up <0.001% of the ingested load [16]. These values are supported by another study on *Gammarus pulex*, showing that the average size of microplastic found in the body and faeces was 65 µm, with the total range extending from 14 to 555 µm [58]. Yet, under field conditions, studies of microplastic in macroinvertebrates, that have analysed the size of the observed particles, report sizes of between 15 and 1,910 µm [4, 59]. In addition, Simmerman and Coleman 2020 [4] found that while the water was dominated by microplastic <50 µm in their study, the macroinvertebrates contained larger sized microplastic particles, primarily between 100 and 330 µm. This reveals a discrepancy between findings from studies performed under lab and field conditions. Microplastic found in the sediment of the River Folla is theoretically edible in terms of the size that is possible to ingest for macroinvertebrates found in this location, albeit in the upper end of size range of preferred food items. Therefore, it cannot be disregarded that had the average plastic size been lower, ingestion by macroinvertebrates might have been higher. It would have been advantageous to analyse smaller particles <50 µm, as these small sized particles have been found to be taken up by freshwater macroinvertebrates (e.g. [60–62]). However, this is also associated with additional analytical challenges which are imposed by methods and instruments for accurately and reliably detecting microplastic concentrations.

Since only a single piece of microplastic was found in the macroinvertebrates collected, a comparison between feeding traits or other variables is not possible. At present, there are too few studies to make conclusions about the differences in microplastic ingestion between different feeding traits, especially given that at present the available results can point in different directions [18, 52, 63–67]. Further study is needed to delve into the potential influence of different variables on the ingestion of microplastic under field conditions, including at locations where higher levels of ingestion are observed.

An additional point to consider is that the microplastic that was found in the sediment is too large to be transferred from the gut to the tissue in these macroinvertebrate species [68]. Thus, the current study represents a snapshot in time of what may have been passing through their relatively short digestive tract, due to their short gut evacuation time [55, 69]. As a result, it is not possible to completely exclude the potential for risk associated with ingestion of microplastic at these sites. Yet, the data indicate that microplastic does not represent a significant risk to these species, exposed to microplastic from this point source.

## Conclusion

This study of microplastic pollution in sediment (75–5,000 µm) in a remote stream near a plastic recycling plant revealed high microplastic concentrations of polyethylene films at two locations downstream of the recycling plant, showing how a plastic recycling plant causes microplastic pollution in a remote area. Apart from pollution originating from the plastic recycling plant, a relatively stable concentration of polypropylene was found at all sites, indicating a diffuse source of this from the catchment, potentially from agricultural activity. This is interesting since agriculture constitutes only a minor area of the catchment; yet, the results point towards mismanagement of plastic waste products resulting in losses to the environment. Despite high sediment concentrations, only one piece of plastic film (50–5,000 µm) was found in all of the 549 macroinvertebrate specimens investigated. This result indicates that microplastic cannot be considered to be a risk in this ecosystem for the studied organisms at this site, and that they do not make up a significant route of transfer to higher trophic levels.

For future field studies, additional sampling sites further upstream from the plastic recycling plant would provide insights into the contribution of polyethylene microplastic from atmospheric transport, as well as other sources in the catchment. In addition, future studies should aim to include more sampling points and increase the resolution, to enhance our understanding of the downstream patterns of contamination associated with a point source.

## Supplementary Information

**Additional file 1.** Combined SI.

## Data Availability

The datasets used and/or analysed during the current study are available from the corresponding author on reasonable request.

## Abbreviations

LOD: Limit of detection
LOQ: Limit of quantification
NaI: Sodium Iodide
