# Supplementary material for: Plastic recycling plant as a point source of microplastics to sediment and macroinvertebrates in a remote stream
Source: Microplast nanoplast. 2022 Dec 8;2(1):26. doi: 10.1186/s43591-022-00045-z (PMC9734615; doi:10.1186/s43591-022-00045-z)
Supplement: Supplementary file 1 — Additional file 1. Combined SI. [file 43591_2022_45_MOESM1_ESM.docx]

Supplementary Information

**Plastic recycling plant as a point source of microplastics to sediment and macroinvertebrates in a remote stream**

Kallenbach, E.M.F., Eriksen, T.E., Hurley, R., Jacobsen, D., Singdahl-Larsen, C. & Friberg, N.

Contents:

4 tables

1 figure

1 dataset

**Table SI1**: List of microplastic studies of freshwater macroinvertebrates.

|  | **Reference** | **Species** | **Setting** |
| --- | --- | --- | --- |
| 1 | (Imhof et al., 2013) | *Lumbriculus variegatus*  *Potamopyrgus antipodarum*  *Gammarus pulex* | Exposure |
| 2 | (Au et al., 2015) | *Hyalella azteca* | Exposure |
| 3 | (Blarer and Burkhardt-Holm, 2016) | *Gammarus fassarum* | Exposure |
| 4 | (Imhof and Laforsch, 2016) | *Potamopyrgus antipodarum* | Exposure |
| 5 | (Beckingham and Ghosh, 2017) | *Lumbriculus variegatus* | Exposure |
| 6 | (Rochman et al., 2017) | *Corbicula fluminea* | Exposure |
| 7 | (Scherer et al., 2017) | *Lumbriculus variegatus*  *Physella acuta*  *Chironomus riparius*  *Gammarus pulex* | Exposure |
| 8 | (Straub et al., 2017) | *Gammarus fassarum* | Exposure |
| 9 | (Guilhermino et al., 2018) | *Corbicula fluminea* | Exposure |
| 10 | (Kim et al., 2018) | *Cybister japonicus* | Exposure |
| 11 | (Magni et al., 2018) | *Dreissena polymorpha* | Exposure |
| 12 | (Murphy and Quinn, 2018) | *Hydra attenuata* | Exposure |
| 13 | (Redondo-Hasselerharm et al., 2018b) | *Gammarus pulex*  *Asellus aquaticus*  *Sphaerium corneum*  *Lumbriculus variegatus*  *Tubifex spp.*  *Hyalella azteca* | Exposure |
| 14 | (Redondo-Hasselerharm et al., 2018a) | *Gammarus pulex* | Exposure |
| 15 | (Vosshage et al., 2018) | *Radix baltica* | Exposure |
| 16 | (Weber et al., 2018) | *Gammarus pulex* | Exposure |
| 17 | (Ziajahromi et al., 2018b) | *Chironomus tepperi* | Exposure |
| 18 | (Al-Jaibachi et al., 2019) | *Culex pipiens* | Exposure |
| 19 | (Lv et al., 2019) | *Procambarus clarkii* | Exposure |
| 20 | (Mateos-Cárdenas et al., 2019) | *Gammarus duebeni* | Exposure |
| 21 | (Silva et al., 2019) | *Chironomus riparius* | Exposure |
| 22 | (Ziajahromi et al., 2019) | *Chironomus tepperi* | Exposure |
| 23 | (Binelli et al., 2020) | *Dreissena polymorpha* | Exposure |
| 24 | (Castro et al., 2020) | *Allonais inaequalis* | Exposure |
| 25 | (Chagas et al., 2020) | *Aphylla williamsoni* | Exposure |
| 26 | (Ehlers et al., 2020) | *Lepidostoma basale* | Exposure |
| 27 | (Fueser et al., 2020) | chironomids | Exposure |
| 28 | (Gambino et al., 2020) | *Dugesia japonica* | Exposure |
| 29 | (Hanslik et al., 2020) | *Chironomus riparius* | Exposure |
| 30 | (Huang et al., 2020a) | *Chironomus sp.* | Esposure |
| 31 | (López-Rojo et al., 2020) | *Sericostoma pyrenaicum* | Exposure |
| 32 | (Mateos-Cárdenas et al., 2020) | *Gammarus duebeni* | Exposure |
| 33 | (Moreschi et al., 2020) | *Anodontites trapesialis* | Exposure |
| 34 | (Pedersen et al., 2020) | *Dreissena bugensis* | Exposure |
| 35 | (Scherer et al., 2020) | *Chironomus riparius* | Exposure |
| 36 | (Silva et al., 2020) | *Lumbriculus variegatus* | Exposure |
| 37 | (Stanković et al., 2020) | *Chironomus riparius* | Exposure |
| 38 | (Yardy and Callaghan, 2020) | *Gammarus pulex* | Exposure |
| 39 | (Gallitelli et al., 2021) | *Odontocerum albicorne*  *Ephemera danica* | Exposure |
| 40 | (Setyorini et al., 2021) | *Chironomus riparius* | Exposure |
| 41 | (Silva et al., 2021) | *Chironomus riparius* | Exposure |
| 42 | (Silva et al., 2022) | 21 taxa | Exposure |
|  |  |  |  |
| 1 | (Su et al., 2016) | *Corbicula fluminea* | *In situ* |
| 2 | (Hurley et al., 2017) | *Tubifex tubifex* | *In situ* |
| 3 | (Nel et al., 2018) | *Chironomus spp.* | *In situ* |
| 4 | (Su et al., 2018) | *Corbicula fluminea* | *In situ* |
| 5 | (Akindele et al., 2019) | *Theodoxus fluviatilis*  *Lanistes varicus*  *Melanoides tuberculata* | *In situ* |
| 6 | (Berglund et al., 2019) | *Anodonta anatina* | *In situ* |
| 7 | (Domogalla-Urbansky et al., 2019) | *Unio pictorum* | *In situ* |
| 8 | (Schessl et al., 2019) | *Dreissena polymorpha*  *Dreissena bugensis* | *In situ* |
| 9 | (Windsor et al., 2019) | Heptageniidae  Baetidae  Hydropsychidae | *In situ* |
| 10 | (Akindele et al., 2020) | *Chironomus Sp.*  *Siphlonurus sp.*  *Lestes viridis* | *In situ* |
| 11 | (Baldwin et al., 2020) | *Dreissena bugensis*  *Corbicula fluminea* | *In situ* |
| 12 | (Dahms et al., 2020) | *Chironomus Sp.* | *In situ* |
| 13 | (Iannilli et al., 2020b) | *Cryptorchestia garbinii* | *In situ* |
| 14 | (Nan et al., 2020) | *Paratya australiensis* | *In situ* |
| 15 | (Pastorino et al., 2020) | *Chironomus Sp.* | *In situ* |
| 16 | (Pazos et al., 2020) | *Limnoperna fortunei* | *In situ* |
| 17 | (Simmerman and Coleman Wasik, 2020) | Heptageniidae  Hydropsychidae  Gammaridae | *In situ* |
| 18 | (Wardlaw and Prosser, 2020) | *Lasmigona costata* | *In situ* |
| 19 | (Doucet et al., 2021) | *Margaritifera margaritifera L.* | *In situ* |
| 20 | (Garcia et al., 2021) | 36 different taxa | *In situ* |
| 21 | (Pastorino et al., 2021) | *Dreissena polymorpha* | *In situ* |
| 22 | (Kallenbach et al., 2022a) | *Dreissena polymorpha* | *In situ* |
|  |  |  |  |
| 1 | (Redondo-Hasselerharm et al., 2020) | Benthic community | *In situ + exposure* |
| 2 | (Stanković et al., 2021) | Benthic community | *In situ + exposure* |
| 3 | (Hoellein et al., 2021) | *Dreissena sp.* | *In situ +*  *exposure* |

**Reference list for Table SI1**

Akindele, E. O., S. M. Ehlers and J. H. Koop (2019). "First empirical study of freshwater microplastics in West Africa using gastropods from Nigeria as bioindicators." Limnologica **78**: 125708.

Akindele, E. O., S. M. Ehlers and J. H. Koop (2020). "Freshwater insects of different feeding guilds ingest microplastics in two Gulf of Guinea tributaries in Nigeria." Environmental Science and Pollution Research **27**(26): 33373-33379.

Al-Jaibachi, R., R. N. Cuthbert and A. Callaghan (2019). "Examining effects of ontogenic microplastic transference on Culex mosquito mortality and adult weight." Science of the Total Environment **651**: 871-876.

Au, S. Y., T. F. Bruce, W. C. Bridges and S. J. Klaine (2015). "Responses of Hyalella azteca to acute and chronic microplastic exposures." Environmental toxicology and chemistry **34**(11): 2564-2572.

Baldwin, A. K., A. R. Spanjer, M. R. Rosen and T. Thom (2020). "Microplastics in Lake Mead national recreation area, USA: occurrence and biological uptake." PloS one **15**(5): e0228896.

Beckingham, B. and U. Ghosh (2017). "Differential bioavailability of polychlorinated biphenyls associated with environmental particles: Microplastic in comparison to wood, coal and biochar." Environmental Pollution **220**: 150-158.

Berglund, E., V. Fogelberg, P. A. Nilsson and J. Hollander (2019). "Microplastics in a freshwater mussel (Anodonta anatina) in Northern Europe." Science of the total environment **697**: 134192.

Binelli, A., L. Pietrelli, S. Di Vito, L. Coscia, M. Sighicelli, C. Della Torre, C. C. Parenti and S. Magni (2020). "Hazard evaluation of plastic mixtures from four Italian subalpine great lakes on the basis of laboratory exposures of zebra mussels." Science of the Total Environment **699**: 134366.

Blarer, P. and P. Burkhardt-Holm (2016). "Microplastics affect assimilation efficiency in the freshwater amphipod Gammarus fossarum." Environmental Science and Pollution Research **23**(23): 23522-23532.

Castro, G. B., A. C. Bernegossi, F. R. Pinheiro, M. C. Felipe and J. J. Corbi (2020). "Effects of Polyethylene Microplastics on Freshwater Oligochaeta Allonais inaequalis (Stephenson, 1911) Under Conventional and Stressful Exposures." Water, Air, & Soil Pollution **231**(9): 1-13.

Dahms, H. T., G. J. van Rensburg and R. Greenfield (2020). "The microplastic profile of an urban African stream." Science of The Total Environment **731**: 138893.

Domogalla-Urbansky, J., P. M. Anger, H. Ferling, F. Rager, A. C. Wiesheu, R. Niessner, N. P. Ivleva and J. Schwaiger (2019). "Raman microspectroscopic identification of microplastic particles in freshwater bivalves (Unio pictorum) exposed to sewage treatment plant effluents under different exposure scenarios." Environmental Science and Pollution Research **26**(2): 2007-2012.

Doucet, C. V., A. L. Labaj and J. Kurek (2021). "Microfiber Content in Freshwater Mussels from Rural Tributaries of the Saint John River, Canada." Water, Air, & Soil Pollution **232**(1): 1-12.

Fueser, H., M.-T. Mueller and W. Traunspurger (2020). "Ingestion of microplastics by meiobenthic communities in small-scale microcosm experiments." Science of The Total Environment **746**: 141276.

Gallitelli, L., A. Cera, G. Cesarini, L. Pietrelli and M. Scalici (2021). "Preliminary indoor evidences of microplastic effects on freshwater benthic macroinvertebrates." Scientific reports **11**(1): 1-11.

Gambino, G., A. Falleni, M. Nigro, A. Salvetti, A. Cecchettini, C. Ippolito, P. Guidi and L. Rossi (2020). "Dynamics of interaction and effects of microplastics on planarian tissue regeneration and cellular homeostasis." Aquatic Toxicology **218**: 105354.

Garcia, F., A. R. de Carvalho, L. Riem-Galliano, L. Tudesque, M. Albignac, A. Ter Halle and J. Cucherousset (2021). "Stable Isotope Insights into Microplastic Contamination within Freshwater Food Webs." Environmental Science & Technology.

Guilhermino, L., L. R. Vieira, D. Ribeiro, A. S. Tavares, V. Cardoso, A. Alves and J. M. Almeida (2018). "Uptake and effects of the antimicrobial florfenicol, microplastics and their mixtures on freshwater exotic invasive bivalve Corbicula fluminea." Science of The Total Environment **622**: 1131-1142.

Hanslik, L., C. Sommer, S. Huppertsberg, S. Dittmar, T. P. Knepper and T. Braunbeck (2020). "Microplastic-associated trophic transfer of benzo (k) fluoranthene in a limnic food web: Effects in two freshwater invertebrates (Daphnia magna, Chironomus riparius) and zebrafish (Danio rerio)." Comparative Biochemistry and Physiology Part C: Toxicology & Pharmacology **237**: 108849.

Hurley, R. R., J. C. Woodward and J. J. Rothwell (2017). "Ingestion of Microplastics by Freshwater Tubifex Worms." Environmental Science & Technology **51**(21): 12844-12851.

Iannilli, V., F. Corami, P. Grasso, F. Lecce, M. Buttinelli and A. Setini (2020). "Plastic abundance and seasonal variation on the shorelines of three volcanic lakes in Central Italy: can amphipods help detect contamination?" Environmental Science and Pollution Research: 1-12.

Imhof, H. K., N. P. Ivleva, J. Schmid, R. Niessner and C. Laforsch (2013). "Contamination of beach sediments of a subalpine lake with microplastic particles." Current Biology **23**(19): R867-R868.

Imhof, H. K. and C. Laforsch (2016). "Hazardous or not–Are adult and juvenile individuals of Potamopyrgus antipodarum affected by non-buoyant microplastic particles?" Environmental pollution **218**: 383-391.

Kim, S. W., D. Kim, Y. Chae and Y.-J. An (2018). "Dietary uptake, biodistribution, and depuration of microplastics in the freshwater diving beetle Cybister japonicus: effects on predacious behavior." Environmental Pollution **242**: 839-844.

López-Rojo, N., J. Pérez, A. Alonso, F. Correa-Araneda and L. Boyero (2020). "Microplastics have lethal and sublethal effects on stream invertebrates and affect stream ecosystem functioning." Environmental Pollution **259**: 113898.

Lv, W., W. Zhou, S. Lu, W. Huang, Q. Yuan, M. Tian, W. Lv and D. He (2019). "Microplastic pollution in rice-fish co-culture system: A report of three farmland stations in Shanghai, China." Science of the Total Environment **652**: 1209-1218.

Magni, S., F. Gagné, C. André, C. Della Torre, J. Auclair, H. Hanana, C. C. Parenti, F. Bonasoro and A. Binelli (2018). "Evaluation of uptake and chronic toxicity of virgin polystyrene microbeads in freshwater zebra mussel Dreissena polymorpha (Mollusca: Bivalvia)." Science of The Total Environment **631**: 778-788.

Mateos-Cárdenas, A., J. O’Halloran, F. N. van Pelt and M. A. Jansen (2020). "Rapid fragmentation of microplastics by the freshwater amphipod Gammarus duebeni (Lillj.)." Scientific reports **10**(1): 1-12.

Mateos-Cárdenas, A., D. T. Scott, G. Seitmaganbetova, N. van Pelt Frank and J. M. AK (2019). "Polyethylene microplastics adhere to Lemna minor (L.), yet have no effects on plant growth or feeding by Gammarus duebeni (Lillj.)." Science of the Total Environment **689**: 413-421.

Murphy, F. and B. Quinn (2018). "The effects of microplastic on freshwater Hydra attenuata feeding, morphology & reproduction." Environmental Pollution **234**: 487-494.

Nan, B., L. Su, C. Kellar, N. J. Craig, M. J. Keough and V. Pettigrove (2020). "Identification of microplastics in surface water and Australian freshwater shrimp Paratya australiensis in Victoria, Australia." Environmental Pollution **259**: 113865.

Nel, H. A., T. Dalu and R. J. Wasserman (2018). "Sinks and sources: Assessing microplastic abundance in river sediment and deposit feeders in an Austral temperate urban river system." Science of the Total Environment **612**: 950-956.

Pastorino, P., E. Pizzul, M. Bertoli, S. Anselmi, M. Kušće, V. Menconi, M. Prearo and M. Renzi (2020). "First insights into plastic and microplastic occurrence in biotic and abiotic compartments, and snow from a high-mountain lake (Carnic Alps)." Chemosphere **265**: 129121.

Pastorino, P., M. Prearo, S. Anselmi, V. Menconi, M. Bertoli, A. Dondo, E. Pizzul and M. Renzi (2021). "Use of the Zebra Mussel Dreissena polymorpha (Mollusca, Bivalvia) as a Bioindicator of Microplastics Pollution in Freshwater Ecosystems: A Case Study from Lake Iseo (North Italy)." Water **13**(4): 434.

Pazos, R. S., F. Spaccesi and N. Gómez (2020). "First record of microplastics in the mussel Limnoperna fortunei." Regional Studies in Marine Science **38**: 101360.

Pedersen, A. F., K. Gopalakrishnan, A. G. Boegehold, N. J. Peraino, J. A. Westrick and D. R. Kashian (2020). "Microplastic ingestion by quagga mussels, Dreissena bugensis, and its effects on physiological processes." Environmental Pollution **260**: 113964.

Redondo-Hasselerharm, P., G. Gort, E. Peeters and A. Koelmans (2020). "Nano-and microplastics affect the composition of freshwater benthic communities in the long term." Science advances **6**(5): eaay4054.

Redondo-Hasselerharm, P. E., V. N. de Ruijter, S. M. Mintenig, A. Verschoor and A. A. Koelmans (2018). "Ingestion and chronic effects of car tire tread particles on freshwater benthic macroinvertebrates." Environmental science & technology **52**(23): 13986-13994.

Redondo-Hasselerharm, P. E., D. Falahudin, E. Peeters and A. A. Koelmans (2018). "Microplastic Effect Thresholds for Freshwater Benthic Macroinvertebrates." Environmental Science & Technology **52**(4): 2278-2286.

Rochman, C. M., J. M. Parnis, M. A. Browne, S. Serrato, E. J. Reiner, M. Robson, T. Young, M. L. Diamond and S. J. Teh (2017). "Direct and indirect effects of different types of microplastics on freshwater prey (Corbicula fluminea) and their predator (Acipenser transmontanus)." PloS one **12**(11): e0187664.

Scherer, C., N. Brennholt, G. Reifferscheid and M. Wagner (2017). "Feeding type and development drive the ingestion of microplastics by freshwater invertebrates." Scientific Reports **7**.

Scherer, C., R. Wolf, J. Völker, F. Stock, N. Brennhold, G. Reifferscheid and M. Wagner (2020). "Toxicity of microplastics and natural particles in the freshwater dipteran Chironomus riparius: Same same but different?" Science of the Total Environment **711**: 134604.

Schessl, M., C. Johns and S. Ashpole (2019). " Microbeads in Sediment, Dreissenid Mussels, and Anurans in the Littoral Zone of the Upper St. Lawrence River, New York." Pollution **5**(1): 41-52.

Setyorini, L., D. Michler-Kozma, B. Sures and F. Gabel (2021). "Transfer and effects of PET microfibers in Chironomus riparius." Science of The Total Environment **757**: 143735.

Silva, C. J., A. L. Machado, D. Campos, A. C. Rodrigues, A. L. P. Silva, A. M. Soares and J. L. Pestana (2022). "Microplastics in freshwater sediments: Effects on benthic invertebrate communities and ecosystem functioning assessed in artificial streams." Science of The Total Environment **804**: 150118.

Silva, C. J., A. L. P. Silva, D. Campos, A. L. Machado, J. L. Pestana and C. Gravato (2021). "Oxidative damage and decreased aerobic energy production due to ingestion of polyethylene microplastics by Chironomus riparius (Diptera) larvae." Journal of Hazardous Materials **402**: 123775.

Silva, C. J., A. L. P. Silva, D. Campos, A. M. Soares, J. L. Pestana and C. Gravato (2020). "Lumbriculus variegatus (oligochaeta) exposed to polyethylene microplastics: biochemical, physiological and reproductive responses." Ecotoxicology and Environmental Safety **207**: 111375.

Silva, C. J., A. L. P. Silva, C. Gravato and J. L. Pestana (2019). "Ingestion of small-sized and irregularly shaped polyethylene microplastics affect Chironomus riparius life-history traits." Science of the Total Environment **672**: 862-868.

Simmerman, C. B. and J. K. Coleman Wasik (2020). "The effect of urban point source contamination on microplastic levels in water and organisms in a cold‐water stream." Limnology and Oceanography Letters **5**(1): 137-146.

Stanković, J., D. Milošević, B. Jovanović, D. Savić‐Zdravković, A. Petrović, M. Raković, N. Stanković and M. S. Piperac (2021). "In situ effects of a microplastic mixture on the community structure of benthic macroinvertebrates in a freshwater pond." Environmental toxicology and chemistry.

Stanković, J., D. Milošević, D. Savić-Zdraković, G. Yalçın, D. Yildiz, M. Beklioğlu and B. Jovanović (2020). "Exposure to a microplastic mixture is altering the life traits and is causing deformities in the non-biting midge Chironomus riparius Meigen (1804)." Environmental Pollution **262**: 114248.

Straub, S., P. E. Hirsch and P. Burkhardt-Holm (2017). "Biodegradable and Petroleum-Based Microplastics Do Not Differ in Their Ingestion and Excretion but in Their Biological Effects in a Freshwater Invertebrate Gammarus fossarum." International journal of environmental research and public health **14**(7): 774.

Su, L., H. Cai, P. Kolandhasamy, C. Wu, C. M. Rochman and H. Shi (2018). "Using the Asian clam as an indicator of microplastic pollution in freshwater ecosystems." Environmental Pollution **234**: 347-355.

Su, L., Y. Xue, L. Li, D. Yang, P. Kolandhasamy, D. Li and H. Shi (2016). "Microplastics in taihu lake, China." Environmental pollution **216**: 711-719.

Vosshage, A. T., T. R. Neu and F. Gabel (2018). "Plastic alters biofilm quality as food resource of the freshwater gastropod Radix balthica." Environmental science & technology **52**(19): 11387-11393.

Wardlaw, C. and R. Prosser (2020). "Investigation of Microplastics in Freshwater Mussels (Lasmigona costata) From the Grand River Watershed in Ontario, Canada." Water, Air, & Soil Pollution **231**(8): 1-14.

Weber, A., C. Scherer, N. Brennholt, G. Reifferscheid and M. Wagner (2018). "PET microplastics do not negatively affect the survival, development, metabolism and feeding activity of the freshwater invertebrate Gammarus pulex." Environmental Pollution **234**: 181-189.

Windsor, F. M., R. M. Tilley, C. R. Tyler and S. J. Ormerod (2019). "Microplastic ingestion by riverine macroinvertebrates." Science of the total environment **646**: 68-74.

Yardy, L. and A. Callaghan (2020). "What the fluff is this?-Gammarus pulex prefer food sources without plastic microfibers." Science of The Total Environment **715**: 136815.

Ziajahromi, S., A. Kumar, P. A. Neale and F. D. Leusch (2019). "Effects of polyethylene microplastics on the acute toxicity of a synthetic pyrethroid to midge larvae (Chironomus tepperi) in synthetic and river water." Science of the total environment **671**: 971-975.

Ziajahromi, S., A. Kumar, P. A. Neale and F. D. L. Leusch (2018). "Environmentally relevant concentrations of polyethylene microplastics negatively impact the survival, growth and emergence of sediment-dwelling invertebrates." Environmental Pollution **236**: 425-431.

**Table SI2:** Total abundance of macroinvertebrates at the three sites in 2019 and 2020. Standard deviation is given in parenthesis.

| **Taxa Group** | **Name** | **St. 1** | **St. 2** | **St. 3** |
| --- | --- | --- | --- | --- |
| Arachnida | Acari indet. Ad. |  |  | 9 (3.9) |
| Coleoptera | Elmis aena Ad. | 3 (1) | 2 (0.6) |  |
| Coleoptera | Elmis aena lv. | 804 (97.2) | 110 (34.3) | 15 (3.9) |
| Coleoptera | Hydraena sp. ad. |  | 7 (2.9) |  |
| Coleoptera | Limnius volckmari ad. |  |  | 4 (1.4) |
| Coleoptera | Oreodytes sp. Ad. |  | 1 (0.5) |  |
| Diptera | Ceratopogonidae Indet. Lv. | 392 (63.5) | 127 (46.4) | 233 (44) |
| Diptera | Chaoborus sp. Lv. |  |  | 1 (0.5) |
| Diptera | Chironomidae Indet. Lv. | 4072 (728.3) | 8244 (3411.4) | 2416 (516) |
| Diptera | Dicranota sp. Lv. | 104 (25.6) | 84 (34.5) | 10 (5) |
| Diptera | Diptera indet. Lv. | 1 (0.5) |  |  |
| Diptera | Empididae Indet. Lv. | 3 (1) | 6 (1) | 2 (1) |
| Diptera | Pediciidae indet. Lv. |  | 18 (5.3) | 7 (2.9) |
| Diptera | Psychodidae indet. Lv. | 20 (4.8) | 9 (2.6) | 12 (6) |
| Diptera | Simuliidae Indet. Lv. | 16 (2.8) | 20 (4.8) | 12 (6) |
| Diptera | Tipulidae Indet. Lv. | 10 (5) | 1 (0.5) | 3 (1) |
| Ephemeroptera | Ameletus inopinatus Lv. | 197 (64.1) | 15 (4.2) | 31 (5.3) |
| Ephemeroptera | Baetidae indet. Lv. | 1984 (521.6) | 1318 (391.5) | 842 (204.8) |
| Ephemeroptera | Baetis muticus Lv. | 287 (81.1) | 21 (8.6) | 14 (7) |
| Ephemeroptera | Baetis muticus/niger Lv. | 42 (17.2) | 4 (0.8) | 14 (4.1) |
| Ephemeroptera | Baetis rhodani Lv. | 1616 (265.3) | 1344 (324) | 692 (109.1) |
| Ephemeroptera | Baetis sp. Lv. | 2528 (577.3) | 1452 (446.9) | 80 (40) |
| Ephemeroptera | Ephemerella aroni Lv. | 1 (0.5) |  |  |
| Ephemeroptera | Ephemerella mucronata Lv. | 3 (1) | 7 (2.1) | 32 (9.8) |
| Ephemeroptera | Ephemeroptera indet. Lv. |  |  | 12 (6) |
| Ephemeroptera | Heptagenia dalecarlica Lv. | 270 (51.8) | 43 (11.2) | 70 (10.5) |
| Ephemeroptera | Heptagenia sp. Lv. | 152 (43.2) | 20 (6) |  |
| Ephemeroptera | Heptageniidae indet. Lv. | 146 (42.7) | 45 (9.5) | 122 (34.6) |
| Ephemeroptera | Leptophlebiidae indet. Lv. |  |  | 3 (1.5) |
| Ephemeroptera | Parameletus sp. Lv. |  | 1 (0.5) | 48 (24) |
| Gastropoda | Gastropoda Indet. |  |  | 2 (1) |
| Gastropoda | Radix balthica | 14 (7) |  |  |
| Gastropoda | Radix labiata/balthica | 1 (0.5) |  |  |
| Gastropoda | Radix sp. | 3 (1.5) |  |  |
| Heteroptera | Corixidae indet. Lv. |  |  | 1 (0.5) |
| Hydrachnidia | Hydrachnidia indet. Ad. | 18 (4.1) | 7 (1.7) | 32 (7.2) |
| Megaloptera | Sialis fuliginosa Lv. | 2 (0.6) |  |  |
| Oligochaeta | Oligochaeta Indet. | 452 (103) | 25 (6.7) | 152 (11.2) |
| Plecoptera | Amphinemura borealis Lv. | 5 (1.5) |  |  |
| Plecoptera | Amphinemura sp. Lv. | 996 (263.5) | 36 (9.5) | 45 (10.8) |
| Plecoptera | Amphinemura standfussi Lv. | 1 (0.5) |  |  |
| Plecoptera | Amphinemura sulcicollis Lv. |  | 1 (0.5) |  |
| Plecoptera | Brachyptera risi Lv. |  | 4 (2) | 1 (0.5) |
| Plecoptera | Capnia atra Lv. | 2 (1) | 28 (14) |  |
| Plecoptera | Capnia pygmaea/atra Lv. |  | 14 (7) | 108 (54) |
| Plecoptera | Capnia sp. Lv. | 459 (222.2) | 1014 (446.5) | 572 (166.9) |
| Plecoptera | Capniidae/Leuctridae indet. Lv. | 958 (297.6) | 58 (18.3) | 84 (7.7) |
| Plecoptera | Chloroperlidae indet. Lv. |  |  | 1 (0.5) |
| Plecoptera | Diura nanseni Lv. | 89 (14.4) | 80 (10.5) | 106 (16.2) |
| Plecoptera | Isoperla grammatica Lv. |  | 16 (5.7) | 3 (1) |
| Plecoptera | Isoperla sp. Lv. | 4 (2) | 17 (4.2) | 2 (0.6) |
| Plecoptera | Leuctra hippopus Lv. | 134 (21.7) | 20 (3.2) | 9 (2.2) |
| Plecoptera | Leuctra nigra Lv. |  | 2 (1) |  |
| Plecoptera | Leuctra sp. Lv. | 2 (1) | 988 (475.4) | 256 (75.3) |
| Plecoptera | Nemoura sp. Lv. |  |  | 1 (0.5) |
| Plecoptera | Nemouridae indet. Lv. | 4 (2) | 4 (2) | 2 (1) |
| Plecoptera | Perlodidae indet. Lv. |  |  | 3 (1.5) |
| Plecoptera | Plecoptera indet. Lv. |  | 18 (9) | 60 (13.2) |
| Plecoptera | Protonemura meyeri Lv. |  | 2 (0.6) |  |
| Plecoptera | Siphonoperla burmeisteri Lv. |  | 14 (3) | 44 (8.4) |
| Plecoptera | Taeniopteryx nebulosa Lv. |  | 17 (4.9) | 2 (1) |
| Trichoptera | Agapetus ochripes Lv. | 21 (8.5) |  | 16 (5.7) |
| Trichoptera | Apatania sp. Lv. | 1848 (154.1) | 18 (2.4) | 28 (6.1) |
| Trichoptera | Apatania stigmatella Lv. | 212 (103.4) |  | 1 (0.5) |
| Trichoptera | Arctopsyche ladogensis Lv. | 58 (17.2) | 37 (7.7) | 30 (5.7) |
| Trichoptera | Athripsodes sp. Lv. |  |  | 1 (0.5) |
| Trichoptera | Chaetopteryx sp. Lv. | 4 (2) |  |  |
| Trichoptera | Chaetopteryx/Annitella Lv. | 10 (5) |  |  |
| Trichoptera | Ecclisopteryx dalecarlica Lv. | 8 () | 6 (1.7) | 5 (1.3) |
| Trichoptera | Glossosomatidae indet. Lv. | 10 (5) |  |  |
| Trichoptera | Hydropsyche angustipennis Lv. | 3 (1.5) |  |  |
| Trichoptera | Hydropsyche newae Lv. | 9 (1.3) |  | 23 (7.6) |
| Trichoptera | Hydropsyche pellucidula Lv. |  |  | 1 (0.5) |
| Trichoptera | Hydropsyche saxonica Lv. | 1 (0.5) |  |  |
| Trichoptera | Hydropsyche sp. Lv. | 8 (1.6) | 3 (1) | 25 (6.7) |
| Trichoptera | Hydroptila sp. Lv. | 306 (62.1) |  |  |
| Trichoptera | Ithytrichia sp. Lv. |  | 1 (0.5) | 22 (5.3) |
| Trichoptera | Lepidostoma hirtum Lv. | 1 (0.5) |  | 20 (6) |
| Trichoptera | Leptoceridae indet. Lv. |  |  | 1 (0.5) |
| Trichoptera | Limnephilidae indet. Lv. | 17 (4) | 1 (0.5) |  |
| Trichoptera | Micrasema setiferum Lv. | 12 (4.8) | 3 (1) | 19 (5.5) |
| Trichoptera | Oxyethira sp. Lv. |  | 3 (0.5) |  |
| Trichoptera | Polycentropus flavomaculatus Lv. | 12 (3.8) | 1 (0.5) | 6 (1.3) |
| Trichoptera | Potamophylax sp. Lv. |  | 1 (0.5) |  |
| Trichoptera | Rhyacophila nubila Lv. | 18 (4.4) | 40 (7.3) | 7 (2.9) |
| Trichoptera | Rhyacophila sp. Lv. | 12 (2.6) | 57 (9.7) | 2 (0.6) |
| Trichoptera | Sericostoma personatum Lv. | 68 (21.4) |  |  |

**Table SI3**: Number of individuals and size of the individuals sampled at the three sites.

|  | **Number of indivuduals** | **Size (cm)** | **Feeding trait** | **Habitat** |
| --- | --- | --- | --- | --- |
| ***Arctopsyche ladogensis*** | St. 1: 24  St. 2: 21  St. 3: 60 | 1.7-2.4 | Predator via net.  Collecter-filterer | Large river riffles, high flow velocities |
| ***Diura nanseni*** | St. 1: 57  St. 2: 105  St. 3: 54 | 1-1.5 | Predator | Velocity <30 cm/s  Shallow water (20-50 cm) |
| ***Baetis rhodani*** | St. 1: 42  St. 2: 138  St. 3: 39 | 0.5-1.1 | grazer /scraper  gatherer/collector | Reophilic (fast moving water 40-90 cm/s), 10-60 cm deep |

**Table SI4**: Microplastic concentrations in sediment in European streams and rivers.

| **Reference** | **Concentration (MP/kg)** |
| --- | --- |
| (Blair, Waldron et al. 2019) | 161–432 |
| (Crew, Gregory-Eaves et al. 2020) | 832 |
| (Frei, Piehl et al. 2019) | 10000-50000 |
| (Guerranti, Cannas et al. 2017) | 45–1069 |
| (Horton, Svendsen et al. 2017) | 660 |
| (Hurley, Woodward et al. 2018) | 2812-70600 |
| (Klein, Worch et al. 2015) | 228-3763 |
| (Leslie, Brandsma et al. 2017) | <68 to 10,500 |
| (Mani, Hauk et al. 2015) | 260 (10) -11070 (600) |
| (Morritt, Stefanoudis et al. 2014) |  |
| (Nel, Dalu et al. 2018) | 160.1 (139.5) |
| (Piehl, Mitterwallner et al. 2019) | 2.92 to 23.30 |
| (Rodrigues, Abrantes et al. 2018) | 2.6-629 |
| (Scherer, Weber et al. 2020) | 9-15962 |
| (Simon-Sánchez, Grelaud et al. 2019) | 2050 (746) |
| (Tibbetts, Krause et al. 2018) | 165 |

**Reference list for Table SI4**

Blair, R. M., S. Waldron, V. R. Phoenix and C. Gauchotte-Lindsay (2019). "Microscopy and elemental analysis characterisation of microplastics in sediment of a freshwater urban river in Scotland, UK." Environmental Science and Pollution Research **26**(12): 12491-12504.

Crew, A., I. Gregory-Eaves and A. Ricciardi (2020). "Distribution, abundance, and diversity of microplastics in the upper St. Lawrence River." Environmental Pollution **260**: 113994.

Frei, S., S. Piehl, B. Gilfedder, M. Löder, J. Krutzke, L. Wilhelm and C. Laforsch (2019). "Occurence of microplastics in the hyporheic zone of rivers." Scientific reports **9**(1): 1-11.

Guerranti, C., S. Cannas, C. Scopetani, P. Fastelli, A. Cincinelli and M. Renzi (2017). "Plastic litter in aquatic environments of Maremma Regional Park (Tyrrhenian Sea, Italy): Contribution by the Ombrone river and levels in marine sediments." Marine pollution bulletin **117**(1-2): 366-370.

Horton, A. A., C. Svendsen, R. J. Williams, D. J. Spurgeon and E. Lahive (2017). "Large microplastic particles in sediments of tributaries of the River Thames, UK–Abundance, sources and methods for effective quantification." Marine pollution bulletin **114**(1): 218-226.

Hurley, R., J. Woodward and J. J. Rothwell (2018). "Microplastic contamination of river beds significantly reduced by catchment-wide flooding." Nature Geoscience **11**(4): 251.

Klein, S., E. Worch and T. P. Knepper (2015). "Occurrence and Spatial Distribution of Microplastics in River Shore Sediments of the Rhine-Main Area in Germany." Environmental Science & Technology **49**(10): 6070-6076.

Leslie, H. A., S. H. Brandsma, M. J. M. van Velzen and A. D. Vethaak (2017). "Microplastics en route: Field measurements in the Dutch river delta and Amsterdam canals, wastewater treatment plants, North Sea sediments and biota." Environment International **101**: 133-142.

Mani, T., A. Hauk, U. Walter and P. Burkhardt-Holm (2015). "Microplastics profile along the Rhine River." Scientific reports **5**: 17988.

Morritt, D., P. V. Stefanoudis, D. Pearce, O. A. Crimmen and P. F. Clark (2014). "Plastic in the Thames: a river runs through it." Marine Pollution Bulletin **78**(1): 196-200.

Nel, H. A., T. Dalu and R. J. Wasserman (2018). "Sinks and sources: Assessing microplastic abundance in river sediment and deposit feeders in an Austral temperate urban river system." Science of the Total Environment **612**: 950-956.

Piehl, S., V. Mitterwallner, E. C. Atwood, M. Bochow and C. Laforsch (2019). "Abundance and distribution of large microplastics (1–5 mm) within beach sediments at the Po River Delta, northeast Italy." Marine pollution bulletin **149**: 110515.

Rodrigues, M., N. Abrantes, F. Gonçalves, H. Nogueira, J. Marques and A. Gonçalves (2018). "Spatial and temporal distribution of microplastics in water and sediments of a freshwater system (Antuã River, Portugal)." Science of The Total Environment **633**: 1549-1559.

Scherer, C., A. Weber, F. Stock, S. Vurusic, H. Egerci, C. Kochleus, N. Arendt, C. Foeldi, G. Dierkes and M. Wagner (2020). "Comparative assessment of microplastics in water and sediment of a large European river." Science of The Total Environment **738**: 139866.

Simon-Sánchez, L., M. Grelaud, J. Garcia-Orellana and P. Ziveri (2019). "River Deltas as hotspots of microplastic accumulation: The case study of the Ebro River (NW Mediterranean)." Science of the total environment **687**: 1186-1196.

Tibbetts, J., S. Krause, I. Lynch and G. H. Sambrook Smith (2018). "Abundance, distribution, and drivers of microplastic contamination in urban river environments." Water **10**(11): 1597.


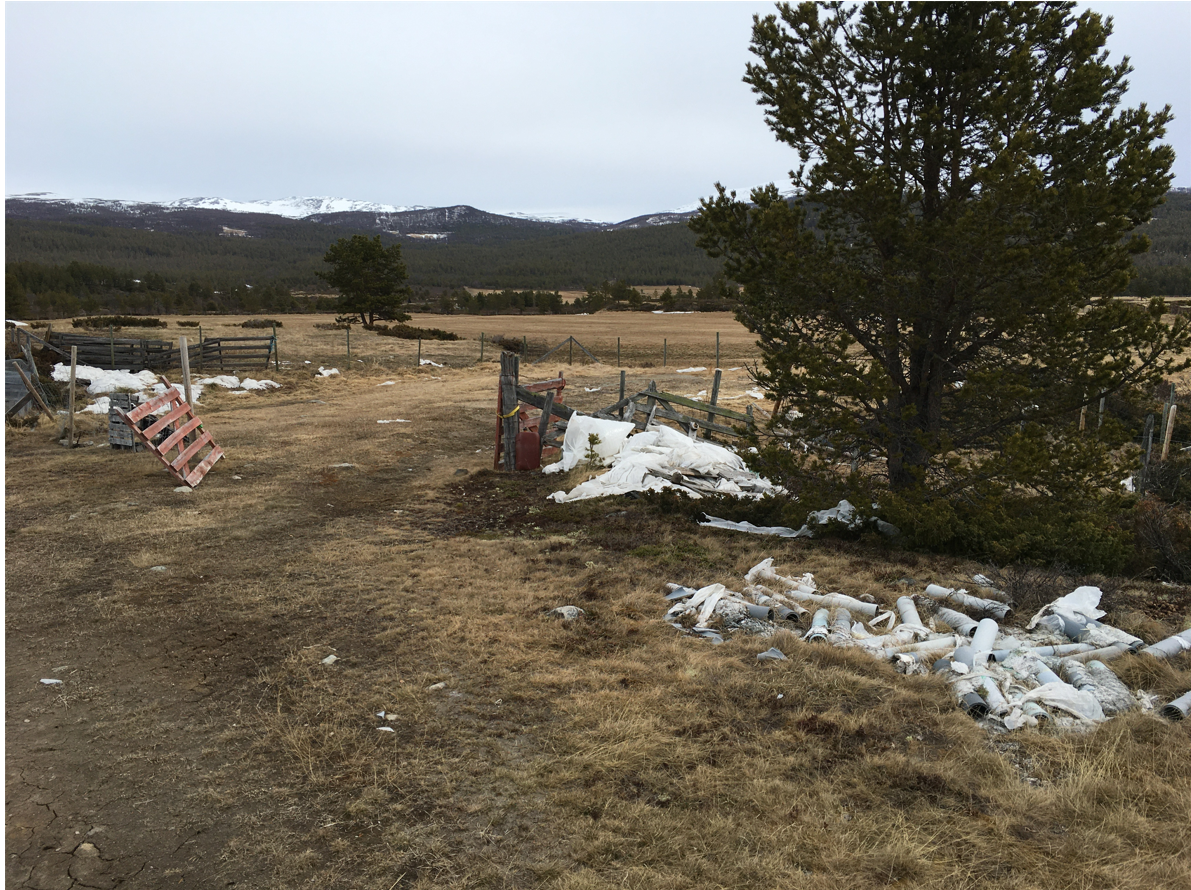


Figure SI1: Plastic waste on the ground next to St. 1.

**Supplementary dataset**

**Table SI5: Results from FTIR analysis**

Summary

|  | **Visual** | **FTIR** |  |  |  |
| --- | --- | --- | --- | --- | --- |
| **F1.1** | 20 | 16 |  |  |  |
| **F1.2** | 30 | 26 |  |  |  |
| **F1.3** | 25 | 19 |  |  |  |
| **F2.1** | 38 | 34 |  |  |  |
| **F2.2** | 32 | 30 |  |  |  |
| **F2.3** | 62 | 59 |  |  |  |
| **F3.1** | 89 | 87 |  |  |  |
| **F3.2** | 42 | 40 |  |  |  |
| **F3.3** | 31 | 29 |  |  |  |
|  |  | 340 |  |  |  |
|  |  |  |  |  |  |
|  |  |  |  |  |  |
|  | **PE** | **PP** | **PTFE** | **Expanded polystyrene** | **Total** |
| **F1.1** | 9 | 6 | 1 | 0 | 16 |
| **F1.2** | 19 | 7 | 0 | 0 | 26 |
| **F1.3** | 9 | 9 | 0 | 1 | 19 |
| **F2.1** | 31 | 3 | 0 | 0 | 34 |
| **F2.2** | 23 | 7 | 0 | 0 | 30 |
| **F2.3** | 41 | 18 | 0 | 0 | 59 |
| **F3.1** | 60 | 27 | 0 | 0 | 87 |
| **F3.2** | 37 | 3 | 0 | 0 | 40 |
| **F3.3** | 28 | 1 | 0 | 0 | 29 |
|  |  |  |  |  | 340 |
|  |  |  |  |  |  |
|  |  |  |  |  |  |
|  | **Longest (µm)** | **Shortest (µm)** | **Average** |  |  |
| **F1.1** | 1127 | 118 | 376 |  |  |
| **F1.2** | 794 | 115 | 345 |  |  |
| **F1.3** | 962 | 150 | 398 |  |  |
| **F2.1** | 3176 | 229 | 1298 |  |  |
| **F2.2** | 1845 | 182 | 805 |  |  |
| **F2.3** | 3589 | 127 | 680 |  |  |
| **F3.1** | 2636 | 125 | 587 |  |  |
| **F3.2** | 4260 | 170 | 1033 |  |  |
| **F3.3** | 2454 | 155 | 806 |  |  |
|  |  |  |  |  |  |
|  |  |  |  |  |  |
|  |  |  |  |  |  |
|  |  |  |  |  |  |
| **Recovery test** |  |  |  |  |  |
|  |  |  |  |  |  |
| **Polymer** | **Shape** | **Colour** | **Size (µm)** | **Added** | **Recovered** |
| PE | bead | white/transparent | 400-450 | 10 | 9 |
| Polyester | fiber | red/orange | 200-700 | 10 | 7 |

Raw data samples

| **Sample ID** | **Extraction_#** | **Particle_#** | **Type** | **Colour** | **Longest (um)** | **Shortest (um)** | **Comment** | **FT-IR match** | **Polymer** | **Comment FT-IR** |
| --- | --- | --- | --- | --- | --- | --- | --- | --- | --- | --- |
| F1.1 | 1 | 1 | Fragment/film | Transparent | 449 | 161 |  | 0.989572 | polyethylene |  |
| F1.1 | 1 | 2 | Fragment/film | Transparent | 239 | 138 |  | 0.98856 | polyethylene |  |
| F1.1 | 1 | 3 | Fragment/film | Transparent | 238 | 135 |  | 0.994317 | polyethylene |  |
| F1.1 | 1 | 4 | Fragment/film | Transparent | 299 | 143 |  | 0.89118 | polytetrafluoroethylene |  |
| F1.1 | 2 | 5 | Fragment/film | Transparent | 391 | 172 |  | 0.989078 | polyethylene |  |
| F1.1 | 2 | 6 | Fragment/film | Transparent | 200 | 86 |  | 0.988558 | polypropylene |  |
| F1.1 | 2 | 7 | Fragment/film | Transparent | 318 | 125 |  | 0.992977 | polyethylene |  |
| F1.1 | 2 | 8 | Fragment/film | Transparent | 243 | 94 |  | 0.982388 | polypropylene |  |
| F1.1 | 2 | 9 | Fragment/film | Transparent | 268 | 122 |  | 0.979936 | polypropylene |  |
| F1.1 | 2 | 10 | Fragment/film | Transparent | 339 | 219 |  | 0.979107 | polypropylene |  |
| F1.1 | 2 | 11 | Fiber | Transparent | 1127 | 15 |  | 0.829776 | alginic acid sodium salt | not plastic |
| F1.1 | 2 | 12 | Fragment/film | Transparent | 194 | 134 |  | 0.990593 | polyethylene |  |
| F1.1 | 2 | 13 | Fragment/film | Transparent | 384 | 111 |  | 0.430558 | algae desmarestia viridis | not plastic |
| F1.1 | 2 | 14 | Fragment/film | Transparent | 195 | 285 |  | 0.986803 | polyethylene |  |
| F1.1 | 2 | 15 | Fragment/film | Transparent | 728 | 40 |  | 0.97496 | cellulose | not plastic |
| F1.1 | 2 | 16 | Fragment/film | Transparent | 123 | 120 |  | 0.986403 | polypropylene |  |
| F1.1 | 2 | 17 | Fragment/film | Transparent | 314 | 46 |  | 0.974216 | polypropylene |  |
| F1.1 | 1 | 18 | Fiber | Transparent | 1080 | 14 |  | 0.973908 | cellulose | not plastic |
| F1.1 | 1 | 19 | Fragment/film | Transparent | 274 | 87 |  | 0.986355 | polyethylene |  |
| F1.1 | 1 | 20 | Fragment/film | Transparent | 118 | 326 |  | 0.991448 | polyethylene |  |
| F2.1 | 1 | 1 | Fragment/film | Transparent | 860 | 21 |  | 0.7766 | polyethylene | looks like it is polyethylene |
| F2.1 | 1 | 2 | Fragment/film | Transparent | 229 | 69 |  | 0.989887 | polyethylene |  |
| F2.1 | 1 | 3 | Fragment/film | White | 1817 | 40 |  | 0.992033 | polyethylene |  |
| F2.1 | 1 | 4 | Fragment/film | White | 688 | 60 |  | 0.985857 | polyethylene |  |
| F2.1 | 1 | 5 | Fragment/film | Transparent | 543 | 206 |  | 0.996676 | polyethylene |  |
| F2.1 | 1 | 6 | Fragment/film | White | 1750 | 34 |  | 0.976276 | polyethylene |  |
| F2.1 | 1 | 7 | Fragment/film | Transparent | 2713 | 97 |  | 0.995092 | polyethylene |  |
| F2.1 | 1 | 8 | Fragment/film | Transparent | 333 | 16 |  | 0.875128 | cellulose | not plastic |
| F2.1 | 1 | 9 | Fiber | Transparent | 884 | 16 |  | 0.949816 | polyethylene |  |
| F2.1 | 1 | 10 | Fragment/film | Transparent | 568 | 131 |  | 0.98533 | polypropylene |  |
| F2.1 | 1 | 11 | Fragment/film | Transparent | 1033 | 16 |  | 0.997425 | polyethylene |  |
| F2.1 | 1 | 12 | Fiber | Transparent | 1223 | 19 |  | 0.851658 | crangon chitin exuvie | not plastic |
| F2.1 | 1 | 13 | Fragment/film | Transparent | 756 | 14 |  | 0.991464 | polyethylene |  |
| F2.1 | 1 | 14 | Fragment/film | Transparent | 2503 | 43 |  | 0.962081 | cellulose | not plastic |
| F2.1 | 1 | 15 | Fragment/film | Transparent | 610 | 185 |  | 0.99304 | polyethylene |  |
| F2.1 | 1 | 16 | Fragment/film | Transparent | 1789 | 188 |  | 0.995189 | polyethylene |  |
| F2.1 | 1 | 17 | Fragment/film | Transparent | 1519 | 58 |  | 0.984617 | polyethylene |  |
| F2.1 | 1 | 18 | Fragment/film | Transparent | 1114 | 112 |  | 0.995588 | polyethylene |  |
| F2.1 | 1 | 19 | Fragment/film | Transparent | 2711 | 138 |  | 0.991869 | polyethylene |  |
| F2.1 | 1 | 20 | Fragment/film | Transparent | 3176 | 117 |  | 0.995824 | polyethylene |  |
| F2.1 | 1 | 21 | Fragment/film | Transparent | 3044 | 678 |  | 0.994266 | polyethylene |  |
| F2.1 | 1 | 22 | Fragment/film | Transparent | 2473 | 299 |  | 0.964991 | polyethylene |  |
| F2.1 | 1 | 23 | Fragment/film | White | 2829 | 178 |  | 0.985431 | polyethylene |  |
| F2.1 | 1 | 24 | Fragment/film | White | 2545 | 193 |  | 0.993397 | polyethylene |  |
| F2.1 | 1 | 25 | Fragment/film | White | 1195 | 24 |  | 0.920411 | polyethylene |  |
| F2.1 | 1 | 26 | Fragment/film | Transparent | 724 | 20 |  | 0.984812 | cellulose | not plastic |
| F2.1 | 2 | 27 | Fragment/film | White | 463 | 67 |  | 0.993897 | polyethylene |  |
| F2.1 | 2 | 28 | Fragment/film | Transparent | 457 | 287 |  | 0.987478 | polyethylene |  |
| F2.1 | 2 | 29 | Fragment/film | Transparent | 1909 | 43 |  | 0.990883 | polyethylene |  |
| F2.1 | 2 | 30 | Fragment/film | Transparent | 337 | 60 |  | 0.99074 | polyethylene |  |
| F2.1 | 2 | 31 | Fragment/film | Transparent | 365 | 111 |  | 0.989504 | polyethylene |  |
| F2.1 | 2 | 32 | Fragment/film | Transparent | 260 | 110 |  | 0.98778 | polyethylene |  |
| F2.1 | 2 | 33 | Fragment/film | White | 1904 | 84 |  | 0.980791 | polyethylene |  |
| F2.1 | 2 | 34 | Fragment/film | White | 2441 | 194 |  | 0.986937 | polyethylene |  |
| F2.1 | 2 | 35 | Fragment/film | Transparent | 745 | 34 |  | 0.98304 | polyethylene |  |
| F2.1 | 2 | 36 | Fragment/film | Transparent | 327 | 207 |  | 0.991712 | polyethylene |  |
| F2.1 | 2 | 37 | Fragment/film | Transparent | 230 | 139 |  | 0.974432 | polypropylene |  |
| F2.1 | 2 | 38 | Fragment/film | Transparent | 292 | 58 |  | 0.974535 | polypropylene |  |
| F3.1 | 1 | 1 | Fragment/film | Transparent | 1188 | 140 |  | 0.965317 | polyethylene |  |
| F3.1 | 1 | 2 | Fragment/film | Transparent | 465 | 160 |  | 0.99515 | polyethylene |  |
| F3.1 | 1 | 3 | Fragment/film | Transparent | 390 | 214 |  | 0.987555 | polyethylene |  |
| F3.1 | 1 | 4 | Fragment/film | White | 571 | 298 |  | 0.950825 | kapok | not plastic |
| F3.1 | 1 | 5 | Fragment/film | Transparent | 540 | 96 |  | 0.933442 | cellulose | not plastic |
| F3.1 | 1 | 6 | Fragment/film | Transparent | 311 | 75 |  | 0.995649 | polyethylene |  |
| F3.1 | 1 | 7 | Fragment/film | Transparent | 298 | 70 |  | 0.981707 | polyethylene |  |
| F3.1 | 1 | 8 | Fragment/film | Transparent | 1055 | 223 |  | 0.996607 | polyethylene |  |
| F3.1 | 1 | 9 | Fragment/film | Transparent | 1939 | 76 |  | 0.996735 | polyethylene |  |
| F3.1 | 1 | 10 | Fragment/film | Transparent | 484 | 337 |  | 0.990966 | polyethylene |  |
| F3.1 | 1 | 11 | Fragment/film | Transparent | 1386 | 277 |  | 0.990189 | polyethylene |  |
| F3.1 | 1 | 12 | Fragment/film | Transparent | 373 | 179 |  | 0.992771 | polyethylene |  |
| F3.1 | 1 | 13 | Fragment/film | Transparent | 364 | 164 |  | 0.991797 | polyethylene |  |
| F3.1 | 1 | 14 | Fragment/film | Transparent | 1147 | 336 |  | 0.988134 | polyethylene |  |
| F3.1 | 1 | 15 | Fragment/film | White | 607 | 329 |  | 0.984733 | polyethylene |  |
| F3.1 | 1 | 16 | Fragment/film | Transparent | 1560 | 69 |  | 0.996642 | polyethylene |  |
| F3.1 | 1 | 17 | Fragment/film | White | 1983 | 58 |  | 0.995732 | polyethylene |  |
| F3.1 | 1 | 18 | Fragment/film | White | 1011 | 35 |  | 0.994245 | polyethylene |  |
| F3.1 | 1 | 19 | Fragment/film | White | 1738 | 50 |  | 0.987595 | polyethylene |  |
| F3.1 | 1 | 20 | Fragment/film | Transparent | 687 | 45 |  | 0.994817 | polyethylene |  |
| F3.1 | 1 | 21 | Fragment/film | Transparent | 609 | 96 |  | 0.997026 | polyethylene |  |
| F3.1 | 1 | 22 | Fragment/film | White | 2040 | 45 |  | 0.995087 | polyethylene |  |
| F3.1 | 2 | 23 | Fragment/film | White | 857 | 88 |  | 0.986869 | polyethylene |  |
| F3.1 | 2 | 24 | Fragment/film | Transparent | 476 | 75 |  | 0.981279 | polypropylene |  |
| F3.1 | 2 | 25 | Fragment/film | Transparent | 283 | 127 |  | 0.99333 | polyethylene |  |
| F3.1 | 2 | 26 | Fragment/film | Transparent | 294 | 117 |  | 0.988515 | polyethylene |  |
| F3.1 | 2 | 27 | Fragment/film | Transparent | 125 | 68 |  | 0.983411 | polypropylene |  |
| F3.1 | 2 | 28 | Fragment/film | Transparent | 146 | 87 |  | 0.99306 | polyethylene |  |
| F3.1 | 2 | 29 | Fragment/film | White | 299 | 86 |  | 0.976037 | polypropylene |  |
| F3.1 | 2 | 30 | Fragment/film | Transparent | 513 | 106 |  | 0.990827 | polyethylene |  |
| F3.1 | 2 | 31 | Fragment/film | Transparent | 320 | 84 |  | 0.986794 | polypropylene |  |
| F3.1 | 2 | 32 | Fragment/film | Transparent | 188 | 85 |  | 0.98028 | polypropylene |  |
| F3.1 | 2 | 33 | Fragment/film | Transparent | 321 | 121 |  | 0.977062 | polypropylene |  |
| F3.1 | 2 | 34 | Fragment/film | Transparent | 252 | 85 |  | 0.986763 | polypropylene |  |
| F3.1 | 2 | 35 | Fragment/film | Transparent | 261 | 149 |  | 0.986289 | polyethylene |  |
| F3.1 | 2 | 36 | Fragment/film | Transparent | 212 | 134 |  | 0.993672 | polyethylene |  |
| F3.1 | 2 | 37 | Fragment/film | White | 307 | 50 |  | 0.989327 | polyethylene |  |
| F3.1 | 2 | 38 | Fragment/film | Transparent | 393 | 122 |  | 0.982715 | polypropylene |  |
| F3.1 | 2 | 39 | Fragment/film | Transparent | 158 | 101 |  | 0.978749 | polypropylene |  |
| F3.1 | 2 | 40 | Fragment/film | Transparent | 185 | 94 |  | 0.989685 | polyethylene |  |
| F3.1 | 2 | 41 | Fragment/film | Transparent | 286 | 100 |  | 0.982675 | polypropylene |  |
| F3.1 | 2 | 42 | Fragment/film | White | 1420 | 33 |  | 0.987049 | polyethylene |  |
| F3.1 | 2 | 43 | Fragment/film | Transparent | 339 | 124 |  | 0.896265 | polyethylene |  |
| F3.1 | 2 | 44 | Fragment/film | Transparent | 216 | 173 |  | 0.984701 | polypropylene |  |
| F3.1 | 2 | 45 | Fragment/film | White | 662 | 154 |  | 0.995875 | polyethylene |  |
| F3.1 | 2 | 46 | Fragment/film | Transparent | 315 | 91 |  | 0.975869 | polypropylene |  |
| F3.1 | 2 | 47 | Fragment/film | Transparent | 233 | 90 |  | 0.974567 | polypropylene |  |
| F3.1 | 2 | 48 | Fragment/film | Transparent | 210 | 106 |  | 0.984514 | polypropylene |  |
| F3.1 | 2 | 49 | Fragment/film | Transparent | 229 | 101 |  | 0.99104 | polyethylene |  |
| F3.1 | 2 | 50 | Fragment/film | Transparent | 186 | 75 |  | 0.986841 | polypropylene |  |
| F3.1 | 2 | 51 | Fragment/film | Transparent | 292 | 103 |  | 0.993969 | polyethylene |  |
| F3.1 | 2 | 52 | Fragment/film | Transparent | 269 | 23 |  | 0.985938 | polypropylene |  |
| F3.1 | 2 | 53 | Fragment/film | Transparent | 187 | 81 |  | 0.990465 | polyethylene |  |
| F3.1 | 2 | 54 | Fragment/film | Transparent | 204 | 92 |  | 0.984062 | polypropylene |  |
| F3.1 | 2 | 55 | Fragment/film | Transparent | 468 | 76 |  | 0.98755 | polypropylene |  |
| F3.1 | 2 | 56 | Fragment/film | Transparent | 182 | 94 |  | 0.979155 | polypropylene |  |
| F3.1 | 2 | 57 | Fragment/film | White | 510 | 154 |  | 0.982885 | polypropylene |  |
| F3.1 | 2 | 58 | Fragment/film | Transparent | 287 | 174 |  | 0.971658 | polypropylene |  |
| F3.1 | 2 | 59 | Fragment/film | Transparent | 507 | 158 |  | 0.99209 | polypropylene |  |
| F3.1 | 2 | 60 | Fragment/film | Transparent | 212 | 82 |  | 0.994232 | polyethylene |  |
| F3.1 | 2 | 61 | Fragment/film | Transparent | 205 | 100 |  | 0.994812 | polyethylene |  |
| F3.1 | 2 | 62 | Fragment/film | Transparent | 241 | 44 |  | 0.994205 | polyethylene |  |
| F3.1 | 2 | 63 | Fragment/film | Transparent | 345 | 82 |  | 0.996286 | polyethylene |  |
| F3.1 | 2 | 64 | Fragment/film | Transparent | 263 | 142 |  | 0.99308 | polyethylene |  |
| F3.1 | 2 | 65 | Fragment/film | Transparent | 145 | 80 |  | 0.989327 | polyethylene |  |
| F3.1 | 2 | 66 | Fragment/film | Transparent | 437 | 234 |  | 0.991001 | polyethylene |  |
| F3.1 | 2 | 67 | Fragment/film | Transparent | 191 | 144 |  | 0.995448 | polyethylene |  |
| F3.1 | 2 | 68 | Fragment/film | White | 851 | 251 |  | 0.992277 | polyethylene |  |
| F3.1 | 2 | 69 | Fragment/film | Transparent | 175 | 91 |  | 0.989861 | polyethylene |  |
| F3.1 | 2 | 70 | Fragment/film | Transparent/white | 277 | 155 |  | 0.978195 | polypropylene |  |
| F3.1 | 2 | 71 | Fragment/film | Transparent | 1103 | 56 |  | 0.986351 | polypropylene |  |
| F3.1 | 2 | 72 | Fragment/film | Transparent | 222 | 188 |  | 0.992948 | polyethylene |  |
| F3.1 | 2 | 73 | Fragment/film | Transparent | 568 | 85 |  | 0.989771 | polyethylene |  |
| F3.1 | 2 | 74 | Fragment/film | White | 537 | 45 |  | 0.995182 | polyethylene |  |
| F3.1 | 2 | 75 | Fragment/film | Transparent/white | 697 | 152 |  | 0.995696 | polyethylene |  |
| F3.1 | 2 | 76 | Fragment/film | Transparent | 993 | 29 |  | 0.979286 | polypropylene |  |
| F3.1 | 2 | 77 | Fragment/film | Transparent | 208 | 67 |  | 0.996443 | polyethylene |  |
| F3.1 | 2 | 78 | Fragment/film | Transparent | 724 | 93 |  | 0.994896 | polyethylene |  |
| F3.1 | 2 | 79 | Fragment/film | Transparent | 2330 | 111 |  | 0.991832 | polyethylene |  |
| F3.1 | 2 | 80 | Fragment/film | Transparent | 487 | 98 |  | 0.988065 | polypropylene |  |
| F3.1 | 2 | 81 | Fragment/film | Transparent | 321 | 54 |  | 0.994323 | polyethylene |  |
| F3.1 | 2 | 82 | Fragment/film | Transparent | 1157 | 112 |  | 0.993994 | polyethylene |  |
| F3.1 | 2 | 83 | Fragment/film | Transparent | 243 | 152 |  | 0.996089 | polyethylene |  |
| F3.1 | 2 | 84 | Fragment/film | Transparent/white | 666 | 215 |  | 0.981001 | polyethylene |  |
| F3.1 | 2 | 85 | Fragment/film | White | 783 | 19 |  | 0.994923 | polyethylene |  |
| F3.1 | 2 | 86 | Fragment/film | White | 2636 | 404 |  | 0.995643 | polyethylene |  |
| F3.1 | 1 | 87 | Fragment/film | White | 951 | 40 |  | 0.966189 | polypropylene |  |
| F3.1 | 2 | 88 | Fragment/film | Transparent/white | 789 | 53 |  | 0.993856 | polyethylene |  |
| F3.1 | 2 | 89 | Fragment/film | White | 197 | 43 |  | 0.993856 | polyethylene |  |
| F1.2 | 1 | 1 | Fragment/film | Transparent | 361 | 109 |  | 0.994181 | polyethylene |  |
| F1.2 | 1 | 2 | Fragment/film | Transparent | 115 | 60 |  | 0.98396 | polypropylene |  |
| F1.2 | 1 | 3 | Fragment/film | Transparent | 387 | 256 |  | 0.993782 | polyethylene |  |
| F1.2 | 1 | 4 | Fragment/film | Transparent | 585 | 86 |  | 0.991325 | polyethylene |  |
| F1.2 | 1 | 5 | Fragment/film | Transparent | 411 | 311 |  | 0.899186 | fur | not plastic |
| F1.2 | 1 | 6 | Fragment/film | Transparent | 368 | 75 |  | 0.985469 | polypropylene |  |
| F1.2 | 1 | 7 | Fragment/film | Transparent | 177 | 149 |  | 0.985924 | polyethylene |  |
| F1.2 | 1 | 8 | Fragment/film | Transparent | 221 | 63 |  | 0.99623 | polyethylene |  |
| F1.2 | 1 | 9 | Fragment/film | Transparent | 744 | 545 |  | 0.990922 | polyethylene |  |
| F1.2 | 2 | 10 | Fragment/film | Transparent | 228 | 95 |  | 0.993038 | polyethylene |  |
| F1.2 | 2 | 11 | Fragment/film | Transparent | 265 | 173 |  | 0.993246 | polyethylene |  |
| F1.2 | 2 | 12 | Fragment/film | Transparent | 397 | 134 |  | 0.990236 | polyethylene |  |
| F1.2 | 2 | 13 | Fragment/film | Transparent | 243 | 162 |  | 0.990998 | polyethylene |  |
| F1.2 | 2 | 14 | Fragment/film | Transparent | 223 | 78 |  | 0.983352 | polyethylene |  |
| F1.2 | 2 | 15 | Fragment/film | Transparent | 215 | 102 |  | 0.986429 | polyethylene |  |
| F1.2 | 2 | 16 | Fragment/film | Transparent | 722 | 61 |  | 0.993833 | polyethylene |  |
| F1.2 | 2 | 17 | Fragment/film | Transparent | 133 | 70 |  | 0.985628 | polypropylene |  |
| F1.2 | 2 | 18 | Fragment/film | Transparent | 498 | 188 |  | 0.987155 | polyethylene |  |
| F1.2 | 2 | 19 | Fragment/film | Transparent | 197 | 146 |  | 0.988977 | polyethylene |  |
| F1.2 | 2 | 20 | Fragment/film | Transparent | 208 | 66 |  | 0.994509 | polyethylene |  |
| F1.2 | 2 | 21 | Fragment/film | Transparent | 378 | 166 |  | 0.964874 | polypropylene |  |
| F1.2 | 2 | 22 | Fragment/film | Transparent | 353 | 215 |  | - | lost | lost |
| F1.2 | 2 | 23 | Fragment/film | Transparent | 794 | 69 |  | 0.992057 | polyethylene |  |
| F1.2 | 2 | 24 | Fragment/film | Transparent | 145 | 71 |  | 0.842763 | alginic acid sodium salt | not plastic |
| F1.2 | 2 | 25 | Fiber | Transparent | 622 | 15 |  | 0.736312 | alginic acid sodium salt | not plastic |
| F1.2 | 2 | 26 | Fragment/film | Transparent | 204 | 59 |  | 0.983009 | polypropylene |  |
| F1.2 | 2 | 27 | Fragment/film | Transparent | 152 | 98 |  | 0.992049 | polyethylene |  |
| F1.2 | 2 | 28 | Fragment/film | Transparent | 182 | 135 |  | 0.994885 | polyethylene |  |
| F1.2 | 2 | 29 | Fragment/film | Transparent | 547 | 134 |  | 0.9724 | polypropylene |  |
| F1.2 | 2 | 30 | Fragment/film | Transparent | 283 | 77 |  | 0.977906 | polypropylene |  |
| F1.3 | 1 | 1 | Fragment/film | Transparent | 944 | 177 |  | 0.993839 | polyethylene |  |
| F1.3 | 1 | 2 | Fragment/film | Transparent | 194 | 164 |  | 0.984376 | polypropylene |  |
| F1.3 | 1 | 3 | Fragment/film | Transparent | 319 | 97 |  | 0.983428 | polypropylene |  |
| F1.3 | 1 | 4 | Fragment/film | Transparent | 355 | 200 |  | 0.98516 | polyethylene |  |
| F1.3 | 1 | 5 | Fragment/film | Transparent | 308 | 41 |  | 0.969997 | cellulose | not plastic |
| F1.3 | 1 | 6 | Fragment/film | Transparent | 435 | 31 |  | 0.993343 | polyethylene |  |
| F1.3 | 2 | 7 | Fragment/film | Transparent | 337 | 149 |  | 0.988732 | polyethylene |  |
| F1.3 | 2 | 8 | Fragment/film | Transparent | 346 | 142 |  | 0.985836 | polypropylene |  |
| F1.3 | 2 | 9 | Fragment/film | Transparent | 419 | 134 |  | 0.979724 | polypropylene |  |
| F1.3 | 2 | 10 | Fragment/film | Transparent | 194 | 183 |  | 0.980161 | polypropylene |  |
| F1.3 | 2 | 11 | Fragment/film | Transparent | 494 | 51 |  | 0.980702 | cellulose | not plastic |
| F1.3 | 2 | 12 | Fragment/film | Transparent | 516 | 190 |  | 0.991931 | polyethylene |  |
| F1.3 | 2 | 13 | Fragment/film | Transparent | 444 | 228 |  | 0.97447 | polypropylene |  |
| F1.3 | 2 | 14 | Fragment/film | Transparent | 265 | 41 |  | 0.993502 | polyethylene |  |
| F1.3 | 2 | 15 | Fragment/film | Transparent | 284 | 35 |  | 0.970042 | cellulose | not plastic |
| F1.3 | 2 | 16 | Fragment/film | Transparent | 297 | 119 |  | 0.993903 | polyethylene |  |
| F1.3 | 2 | 17 | Fragment/film | Transparent | 336 | 122 |  | 0.985454 | polypropylene |  |
| F1.3 | 2 | 18 | Fragment/film | Transparent | 150 | 44 |  | - | lost | lost |
| F1.3 | 2 | 19 | Fragment/film | Transparent | 355 | 150 |  | 0.989387 | polyethylene |  |
| F1.3 | 2 | 20 | Fragment/film | Transparent | 492 | 51 |  | 0.825859 | alginic acid sodium salt | not plastic |
| F1.3 | 2 | 21 | Fragment/film | Transparent | 374 | 196 |  | 0.976709 | polypropylene |  |
| F1.3 | 2 | 22 | Fragment/film | Transparent | 962 | 31 |  | 0.960459 | polyethylene |  |
| F1.3 | 2 | 23 | Fragment/film | Transparent | 259 | 31 |  | 0.98068 | polypropylene |  |
| F1.3 | 2 | 24 | Fragment/film | Transparent | 437 | 31 |  | 0.940598 | cellulose | not plastic |
| F1.3 | 2 | 25 | Fragment/film | Transparent | 434 | 92 |  | 0.987639 | expanded polystyrene |  |
| F2.2 | 1 | 1 | Fragment/film | Transparent | 924 | 111 |  | 0.994073 | polyethylene |  |
| F2.2 | 1 | 2 | Fragment/film | Transparent | 1410 | 183 |  | 0.989015 | polyethylene |  |
| F2.2 | 1 | 3 | Fragment/film | Transparent | 1581 | 54 |  | 0.994092 | polyethylene |  |
| F2.2 | 1 | 4 | Fragment/film | Transparent | 1729 | 231 |  | 0.997583 | polyethylene |  |
| F2.2 | 1 | 5 | Fragment/film | White | 520 | 207 |  | 0.989448 | polyethylene |  |
| F2.2 | 1 | 6 | Fragment/film | Transparent | 947 | 68 |  | 0.941532 | wood | not plastic |
| F2.2 | 1 | 7 | Fragment/film | Transparent | 364 | 117 |  | 0.994285 | polyethylene |  |
| F2.2 | 1 | 8 | Fragment/film | White | 488 | 106 |  | 0.99057 | polyethylene |  |
| F2.2 | 1 | 9 | Fragment/film | Transparent | 767 | 163 |  | 0.998393 | polyethylene |  |
| F2.2 | 1 | 10 | Fragment/film | Transparent | 806 | 59 |  | 0.994006 | polyethylene |  |
| F2.2 | 1 | 11 | Fragment/film | White | 670 | 52 |  | 0.987934 | polyethylene |  |
| F2.2 | 1 | 12 | Fragment/film | White | 742 | 21 |  | 0.985747 | polyethylene |  |
| F2.2 | 1 | 13 | Fragment/film | White | 1845 | 75 |  | 0.98983 | polyethylene |  |
| F2.2 | 1 | 14 | Fragment/film | White | 302 | 18 |  | 0.992366 | polyethylene |  |
| F2.2 | 1 | 15 | Fragment/film | White | 413 | 13 |  | 0.909832 | jute | not plastic |
| F2.2 | 1 | 16 | Fragment/film | White | 942 | 31 |  | 0.986028 | polyethylene |  |
| F2.2 | 1 | 17 | Fragment/film | White | 558 | 31 |  | 0.986592 | polyethylene |  |
| F2.2 | 1 | 18 | Fragment/film | White | 196 | 43 |  | 0.937194 | polypropylene |  |
| F2.2 | 2 | 19 | Fragment/film | Transparent | 245 | 66 |  | 0.973952 | polypropylene |  |
| F2.2 | 2 | 20 | Fragment/film | Transparent | 1825 | 59 |  | 0.988339 | polyethylene |  |
| F2.2 | 2 | 21 | Fragment/film | White | 1185 | 211 |  | 0.993705 | polyethylene |  |
| F2.2 | 2 | 22 | Fragment/film | Transparent | 1604 | 33 |  | 0.992684 | polyethylene |  |
| F2.2 | 2 | 23 | Fragment/film | Transparent | 418 | 48 |  | 0.981127 | polypropylene |  |
| F2.2 | 2 | 24 | Fragment/film | White | 654 | 62 |  | 0.990612 | polyethylene |  |
| F2.2 | 2 | 25 | Fragment/film | Transparent | 564 | 35 |  | 0.921317 | polyethylene |  |
| F2.2 | 2 | 26 | Fragment/film | Transparent | 1013 | 187 |  | 0.977518 | polyethylene |  |
| F2.2 | 2 | 27 | Fragment/film | Transparent/white | 746 | 131 |  | 0.996448 | polyethylene |  |
| F2.2 | 2 | 28 | Fragment/film | Transparent/white | 1001 | 39 |  | 0.99224 | polyethylene |  |
| F2.2 | 2 | 29 | Fragment/film | Transparent/white | 182 | 108 |  | 0.977162 | polypropylene |  |
| F2.2 | 2 | 30 | Fragment/film | Transparent/white | 388 | 114 |  | 0.973695 | polypropylene |  |
| F2.2 | 2 | 31 | Fragment/film | Transparent | 300 | 118 |  | 0.971749 | polypropylene |  |
| F2.2 | 2 | 32 | Fragment/film | Transparent/white | 453 | 369 |  | 0.971702 | polypropylene |  |
| F2.3 | 1 | 1 | Fragment/film | Transparent | 484 | 282 |  | 0.991598 | polyethylene |  |
| F2.3 | 1 | 2 | Fragment/film | Transparent | 3123 | 174 |  | 0.994571 | polyethylene |  |
| F2.3 | 1 | 3 | Fragment/film | Transparent | 542 | 71 |  | 0.997047 | polyethylene |  |
| F2.3 | 1 | 4 | Fragment/film | Transparent | 1501 | 92.00 |  | 0.995308 | polyethylene |  |
| F2.3 | 1 | 5 | Fragment/film | White/green | 805 | 230 |  | 0.99167 | polyethylene |  |
| F2.3 | 1 | 6 | Fragment/film | Transparent | 1319 | 314 |  | 0.989153 | polyethylene |  |
| F2.3 | 1 | 7 | Fragment/film | White | 994 | 296 |  | 0.993257 | polyethylene |  |
| F2.3 | 1 | 8 | Fragment/film | White | 2092 | 141 |  | 0.989044 | polyethylene |  |
| F2.3 | 1 | 9 | Fragment/film | Transparent | 499 | 372 |  | 0.989423 | polyethylene |  |
| F2.3 | 2 | 10 | Fragment/film | Transparent | 3589 | 131 |  | 0.993921 | polyethylene |  |
| F2.3 | 2 | 11 | Fragment/film | Transparent | 249 | 138 |  | 0.98128 | polypropylene |  |
| F2.3 | 2 | 12 | Fragment/film | Transparent | 887 | 36 |  | 0.990656 | polyethylene |  |
| F2.3 | 2 | 13 | Fragment/film | Transparent | 575 | 81 |  | 0.997628 | polyethylene |  |
| F2.3 | 2 | 14 | Fragment/film | Transparent | 373 | 95 |  | 0.987849 | polypropylene |  |
| F2.3 | 2 | 15 | Fragment/film | Transparent | 264 | 169 |  | 0.983343 | polypropylene |  |
| F2.3 | 2 | 16 | Fragment/film | Transparent/white | 422 | 99 |  | 0.993796 | polyethylene |  |
| F2.3 | 2 | 17 | Fragment/film | Transparent | 414 | 161 |  | 0.990989 | polyethylene |  |
| F2.3 | 2 | 18 | Fragment/film | Transparent | 472 | 25 |  | 0.991021 | polyethylene |  |
| F2.3 | 2 | 19 | Fragment/film | Transparent | 406 | 51 |  | 0.953806 | down | not plastic |
| F2.3 | 2 | 20 | Fragment/film | Transparent | 277 | 96 |  | 0.99021 | polyethylene |  |
| F2.3 | 2 | 21 | Fragment/film | Transparent | 268 | 25 |  | 0.994188 | polyethylene |  |
| F2.3 | 2 | 22 | Fragment/film | Transparent | 209 | 80 |  | 0.990444 | polyethylene |  |
| F2.3 | 2 | 23 | Fragment/film | Transparent | 221 | 71 |  | 0.991499 | polyethylene |  |
| F2.3 | 2 | 24 | Fragment/film | Transparent | 270 | 180 |  | 0.976956 | polypropylene |  |
| F2.3 | 2 | 25 | Fragment/film | Transparent | 373 | 227 |  | 0.983532 | polypropylene |  |
| F2.3 | 2 | 26 | Fragment/film | Transparent | 910 | 56 |  | 0.996558 | polyethylene |  |
| F2.3 | 2 | 27 | Fragment/film | Transparent/white | 1468 | 32 |  | 0.99006 | polyethylene |  |
| F2.3 | 2 | 28 | Fragment/film | Transparent | 692 | 26 |  | 0.711109 | alginic acid sodium salt | not plastic |
| F2.3 | 2 | 29 | Fragment/film | Transparent | 176 | 100 |  | 0.990606 | polyethylene |  |
| F2.3 | 2 | 30 | Fragment/film | Transparent | 375 | 308 |  | 0.973259 | polypropylene |  |
| F2.3 | 2 | 31 | Fragment/film | Transparent | 280 | 192 |  | 0.979392 | polypropylene |  |
| F2.3 | 2 | 32 | Fragment/film | Transparent | 402 | 56 |  | 0.977254 | polypropylene |  |
| F2.3 | 2 | 33 | Fragment/film | Transparent/white | 334 | 106 |  | 0.979684 | polyethylene |  |
| F2.3 | 2 | 34 | Fragment/film | Transparent | 914 | 314 |  | 0.99722 | polyethylene |  |
| F2.3 | 2 | 35 | Fragment/film | Transparent/white | 341 | 246 |  | 0.969724 | polypropylene |  |
| F2.3 | 2 | 36 | Fragment/film | Transparent | 490 | 20 |  | 0.984488 | polyethylene |  |
| F2.3 | 2 | 37 | Fragment/film | Transparent | 595 | 41 |  | 0.978244 | polypropylene |  |
| F2.3 | 2 | 38 | Fragment/film | Transparent | 232 | 80 |  | 0.973626 | polypropylene |  |
| F2.3 | 2 | 39 | Fragment/film | Transparent | 418 | 100 |  | 0.992571 | polyethylene |  |
| F2.3 | 2 | 40 | Fragment/film | Transparent | 1599 | 427 |  | 0.997673 | polyethylene |  |
| F2.3 | 2 | 41 | Fragment/film | Transparent | 552 | 52 |  | 0.972587 | polypropylene |  |
| F2.3 | 2 | 42 | Fragment/film | Transparent | 288 | 105 |  | 0.994193 | polyethylene |  |
| F2.3 | 2 | 43 | Fragment/film | Transparent | 276 | 137 |  | 0.978732 | polypropylene |  |
| F2.3 | 2 | 44 | Fragment/film | Transparent | 437 | 90 |  | 0.978151 | polypropylene |  |
| F2.3 | 2 | 45 | Fragment/film | Transparent | 354 | 116 |  | 0.994045 | polyethylene |  |
| F2.3 | 2 | 46 | Fragment/film | Transparent | 269 | 108 |  | 0.979702 | polypropylene |  |
| F2.3 | 2 | 47 | Fragment/film | Transparent | 410 | 202 |  | 0.988415 | polyethylene |  |
| F2.3 | 2 | 48 | Fragment/film | Transparent | 214 | 59 |  | 0.990313 | polyethylene |  |
| F2.3 | 2 | 49 | Fragment/film | Transparent/white | 2155 | 60 |  | 0.984752 | polyethylene |  |
| F2.3 | 2 | 50 | Fragment/film | Transparent | 956 | 32 |  | 0.992775 | polyethylene |  |
| F2.3 | 2 | 51 | Fragment/film | Transparent | 332 | 317 |  | 0.982197 | polypropylene |  |
| F2.3 | 2 | 52 | Fragment/film | Transparent | 157 | 115 |  | 0.983586 | polypropylene |  |
| F2.3 | 2 | 53 | Fragment/film | Transparent | 1075 | 84 |  | 0.989663 | polyethylene |  |
| F2.3 | 2 | 54 | Fragment/film | Transparent/white | 545 | 52 |  | 0.977737 | polyethylene |  |
| F2.3 | 1 | 55 | Fragment/film | White | 1124 | 27 |  | 0.996013 | polyethylene |  |
| F2.3 | 1 | 56 | Fragment/film | Transparent | 921 | 15 |  | 0.934884 | wood | not plastic |
| F2.3 | 1 | 57 | Fragment/film | Transparent/white | 496 | 149 |  | 0.996189 | polyethylene |  |
| F2.3 | 1 | 58 | Fragment/film | White | 759 | 32 |  | 0.982809 | polyethylene |  |
| F2.3 | 2 | 59 | Fragment/film | Transparent | 127 | 25 |  | 0.986966 | polyethylene |  |
| F2.3 | 2 | 60 | Fragment/film | Transparent | 241 | 84 |  | 0.993608 | polyethylene |  |
| F2.3 | 2 | 61 | Fragment/film | Transparent/white | 261 | 110 |  | 0.978025 | polypropylene |  |
| F2.3 | 2 | 62 | Fragment/film | Transparent/white | 362 | 41 |  | 0.983975 | polyethylene |  |
| F3.2 | 1 | 1 | Fragment/film | White | 4260 | 220 |  | 0.984696 | polyethylene |  |
| F3.2 | 1 | 2 | Fragment/film | Transparent | 2029 | 208 |  | 0.988843 | polyethylene |  |
| F3.2 | 1 | 3 | Fragment/film | White | 738 | 46 |  | 0.98868 | polyethylene |  |
| F3.2 | 1 | 4 | Fragment/film | Transparent | 1665 | 191 |  | 0.996455 | polyethylene |  |
| F3.2 | 1 | 5 | Fragment/film | Transparent | 541 | 163 |  | 0.992191 | polyethylene |  |
| F3.2 | 1 | 6 | Fragment/film | Transparent | 1753 | 220 |  | 0.990633 | polyethylene |  |
| F3.2 | 1 | 7 | Fiber | Transparent | 2828 | 23 |  | 0.976805 | polyethylene |  |
| F3.2 | 1 | 8 | Fragment/film | White | 2267 | 142 |  | 0.994726 | polyethylene |  |
| F3.2 | 1 | 9 | Fragment/film | Transparent | 1140 | 115 |  | 0.991323 | polyethylene |  |
| F3.2 | 1 | 10 | Fragment/film | Transparent | 911 | 189 |  | 0.996001 | polyethylene |  |
| F3.2 | 1 | 11 | Fragment/film | Transparent | 407 | 100 |  | 0.995278 | polyethylene |  |
| F3.2 | 2 | 12 | Fragment/film | Transparent | 3369 | 99 |  | 0.989383 | polyethylene |  |
| F3.2 | 2 | 13 | Fragment/film | Transparent | 488 | 181 |  | 0.991736 | polyethylene |  |
| F3.2 | 2 | 14 | Fragment/film | Transparent | 232 | 54 |  | 0.996042 | polyethylene |  |
| F3.2 | 2 | 15 | Fragment/film | Transparent | 336 | 200 |  | 0.989907 | polyethylene |  |
| F3.2 | 2 | 16 | Fragment/film | Transparent | 440 | 157 |  | 0.990283 | polyethylene |  |
| F3.2 | 2 | 17 | Fragment/film | White | 1692 | 84 |  | 0.990796 | polyethylene |  |
| F3.2 | 2 | 18 | Fragment/film | Transparent | 229 | 191 |  | 0.988566 | polyethylene |  |
| F3.2 | 2 | 19 | Fragment/film | Transparent | 302 | 48 |  | 0.984427 | polypropylene |  |
| F3.2 | 2 | 20 | Fragment/film | White | 339 | 77 |  | 0.993065 | polyethylene |  |
| F3.2 | 2 | 21 | Fragment/film | Transparent | 336 | 68 |  | 0.984795 | polyethylene |  |
| F3.2 | 2 | 22 | Fragment/film | White | 362 | 204 |  | - | lost | lost |
| F3.2 | 2 | 23 | Fragment/film | Transparent | 170 | 75 |  | 0.982597 | polypropylene |  |
| F3.2 | 2 | 24 | Fragment/film | White | 404 | 74 |  | 0.988476 | polyethylene |  |
| F3.2 | 2 | 25 | Fragment/film | Transparent | 625 | 95 |  | 0.993813 | polyethylene |  |
| F3.2 | 2 | 26 | Fragment/film | White | 901 | 78 |  | 0.990948 | polyethylene |  |
| F3.2 | 2 | 27 | Fragment/film | Transparent | 3297 | 104 |  | 0.993647 | polyethylene |  |
| F3.2 | 2 | 28 | Fragment/film | Transparent | 331 | 214 |  | 0.991351 | polyethylene |  |
| F3.2 | 2 | 29 | Fragment/film | Transparent | 290 | 203 |  | 0.975794 | polypropylene |  |
| F3.2 | 2 | 30 | Fragment/film | Transparent | 610 | 117 |  | 0.991279 | polyethylene |  |
| F3.2 | 1 | 31 | Fragment/film | Transparent | 359 | 117 |  | 0.993371 | polyethylene |  |
| F3.2 | 1 | 32 | Fragment/film | White | 579 | 357 |  | 0.989782 | polyethylene |  |
| F3.2 | 1 | 33 | Fragment/film | White | 394 | 110 |  | 0.995481 | polyethylene |  |
| F3.2 | 1 | 34 | Fragment/film | Transparent | 879 | 172 |  | 0.99743 | polyethylene |  |
| F3.2 | 1 | 35 | Fragment/film | Transparent | 1386 | 55 |  | 0.977213 | cellulose | not plastic |
| F3.2 | 1 | 36 | Fragment/film | White | 473 | 129 |  | 0.991491 | polyethylene |  |
| F3.2 | 1 | 37 | Fragment/film | White | 1468 | 50 |  | 0.993031 | polyethylene |  |
| F3.2 | 1 | 38 | Fragment/film | Transparent | 513 | 250 |  | 0.990924 | polyethylene |  |
| F3.2 | 1 | 39 | Fragment/film | White | 386 | 114 |  | 0.994061 | polyethylene |  |
| F3.2 | 1 | 40 | Fragment/film | Transparent/white | 809 | 79 |  | 0.993673 | polyethylene |  |
| F3.2 | 1 | 41 | Fragment/film | White | 1441 | 65 |  | 0.990191 | polyethylene |  |
| F3.2 | 1 | 42 | Fragment/film | White | 1445 | 55 |  | 0.993735 | polyethylene |  |
| F3.3 | 1 | 1 | Fragment/film | Transparent | 1792 | 100 |  | 0.982881 | polyethylene |  |
| F3.3 | 1 | 2 | Fragment/film | Transparent | 1785 | 42 |  | 0.994444 | polyethylene |  |
| F3.3 | 1 | 3 | Fragment/film | Transparent | 304 | 186 |  | 0.996771 | polyethylene |  |
| F3.3 | 1 | 4 | Fragment/film | Transparent | 519 | 239 |  | 0.99317 | polyethylene |  |
| F3.3 | 1 | 5 | Fragment/film | Transparent | 1601 | 346 |  | 0.991352 | polyethylene |  |
| F3.3 | 1 | 6 | Fragment/film | Transparent | 590 | 133 |  | 0.997515 | polyethylene |  |
| F3.3 | 1 | 7 | Fragment/film | Transparent | 1358 | 30 |  | 0.9954 | polyethylene |  |
| F3.3 | 1 | 8 | Fragment/film | Transparent | 556 | 115 |  | 0.992658 | polyethylene |  |
| F3.3 | 1 | 9 | Fragment/film | Transparent | 528 | 149 |  | 0.99736 | polyethylene |  |
| F3.3 | 1 | 10 | Fragment/film | Transparent | 615 | 130 |  | 0.991202 | polyethylene |  |
| F3.3 | 1 | 11 | Fragment/film | Transparent | 405 | 83 |  | 0.96901 | down | not plastic |
| F3.3 | 1 | 12 | Fragment/film | Transparent | 506 | 165 |  | 0.871065 | polyethylene |  |
| F3.3 | 1 | 13 | Fragment/film | Transparent | 2454 | 574 |  | 0.988618 | polyethylene |  |
| F3.3 | 1 | 14 | Fragment/film | Transparent | 2438 | 442 |  | 0.996182 | polyethylene |  |
| F3.3 | 2 | 15 | Fragment/film | Transparent | 928 | 65 |  | 0.992527 | polyethylene |  |
| F3.3 | 2 | 16 | Fragment/film | Transparent | 326 | 215 |  | 0.980215 | polyethylene |  |
| F3.3 | 2 | 17 | Fragment/film | Transparent | 446 | 119 |  | 0.988765 | polyethylene |  |
| F3.3 | 2 | 18 | Fragment/film | Transparent | 613 | 141 |  | 0.992919 | polyethylene |  |
| F3.3 | 2 | 19 | Fragment/film | Transparent | 267 | 128 |  | 0.992416 | polyethylene |  |
| F3.3 | 1 | 20 | Fragment/film | White | 891 | 345 |  | 0.993209 | polyethylene |  |
| F3.3 | 1 | 21 | Fragment/film | Transparent/white | 915 | 75 |  | 0.924433 | cellulose | not plastic |
| F3.3 | 1 | 22 | Fragment/film | Transparent/white | 496 | 87 |  | 0.986213 | polyethylene |  |
| F3.3 | 1 | 23 | Fragment/film | Transparent/white | 343 | 92 |  | 0.992855 | polyethylene |  |
| F3.3 | 1 | 24 | Fragment/film | Transparent/white | 2178 | 18 |  | 0.985839 | polyethylene |  |
| F3.3 | 2 | 25 | Fragment/film | White/grey | 231 | 68 |  | 0.991333 | polyethylene |  |
| F3.3 | 2 | 26 | Fragment/film | Transparent | 155 | 54 |  | 0.988617 | polypropylene |  |
| F3.3 | 2 | 27 | Fragment/film | White | 261 | 32 |  | 0.992009 | polyethylene |  |
| F3.3 | 2 | 28 | Fragment/film | Transparent | 277 | 100 |  | 0.993261 | polyethylene |  |
| F3.3 | 2 | 29 | Fragment/film | Transparent/white | 685 | 45 |  | 0.9884 | polyethylene |  |
| F3.3 | 2 | 30 | Fragment/film | Transparent/white | 290 | 169 |  | 0.995455 | polyethylene |  |
| F3.3 | 2 | 31 | Fragment/film | Transparent/white | 258 | 141 |  | 0.991014 | polyethylene |  |

Raw data blanks

| **Sample ID** | **Extraction_#** | **Particle_#** | **Type** | **Colour** | **Longest (um)** | **Shortest (um)** | **Comment** | **FT-IR match** | **Polymer** | **Comment FT-IR** |
| --- | --- | --- | --- | --- | --- | --- | --- | --- | --- | --- |
| Blank1 | 1 | - | - | - | - | - | - | - | - | - |
| Blank1 | 2 | - | - | - | - | - | - | - | - | - |
| Blank2 | 1 | - | - | - | - | - | - | - | - | - |
| Blank2 | 2 | - | - | - | - | - | - | - | - | - |
| Blank3 | 1 | 1 | Fragment/film | Transparent | 537 | 32 |  |  |  |  |
| Blank3 | 2 | - | - | - | - | - | - | - | - | - |
